# Differences in elongation of very long chain fatty acids and fatty acid metabolism between triple-negative and hormone receptor-positive breast cancer

**DOI:** 10.1186/s12885-017-3554-4

**Published:** 2017-08-29

**Authors:** Yuji Yamashita, Shin Nishiumi, Seishi Kono, Shintaro Takao, Takeshi Azuma, Masaru Yoshida

**Affiliations:** 1 Division of Breast and Endocrine Surgery, Department of Surgery, Kobe University Graduate School of Medicine, 7-5-1 Kusunoki-cho, Chuo-ku, Kobe, Hyogo 650-0017 Japan; 2 Division of Gastroenterology, Department of Internal Medicine, Kobe University Graduate School of Medicine, 7-5-1 Kusunoki-cho, Chuo-ku, Kobe, Hyogo 650-0017 Japan; 3 Division of Metabolomics Research, Department of Internal Related, Kobe University Graduate School of Medicine, 7-5-1 Kusunoki-cho, Chuo-ku, Kobe, Hyogo 650-0017 Japan; 4 AMED-CREST, AMED, 7-5-1 Kusunoki-cho, Chuo-ku, Kobe, Hyogo 650-0017 Japan

**Keywords:** Elongation of very long chain fatty acids, Fatty acid metabolism, Triple-negative breast cancer, ER-positive PgR-positive, HER2-negative breast cancer, Elongases

## Abstract

**Background:**

Triple-negative breast cancer (TN) is more aggressive than other subtypes of breast cancer and has a lower survival rate. Furthermore, detailed biological information about the disease is lacking. This study investigated characteristics of metabolic pathways in TN.

**Methods:**

We performed the metabolome analysis of 74 breast cancer tissues and the corresponding normal breast tissues using LC/MS. Furthermore, we classified the breast cancer tissues into ER-positive, PgR-positive, HER2-negative breast cancer (EP+H-) and TN, and then the differences in their metabolic pathways were investigated. The RT-PCR and immunostaining were carried out to examine the expression of ELOVL1, 2, 3, 4, 5, 6, and 7.

**Results:**

We identified 142 of hydrophilic metabolites and 278 of hydrophobic lipid metabolites in breast tissues. We found the differences between breast cancer and normal breast tissues in choline metabolism, glutamine metabolism, lipid metabolism, and so on. Most characteristic of comparison between EP+H- and TN were differences in fatty acid metabolism was which were related to the elongation of very long chain fatty acids were detected between TN and EP+H-. Real-time RT-PCR showed that the mRNA expression levels of ELOVL1, 5, and 6 were significantly upregulated by 8.5-, 4.6- and 7.0-fold, respectively, in the TN tumors compared with their levels in the corresponding normal breast tissue samples. Similarly, the mRNA expression levels of ELOVL1, 5, and 6 were also significantly higher in the EP+H- tissues than in the corresponding normal breast tissues (by 4.9-, 3.4-, and 2.1-fold, respectively). The mRNA expression level of ELOVL6 was 2.6-fold higher in the TN tumors than in the EP+H- tumors. During immunostaining, the TN and EP+H- tumors demonstrated stronger ELOVL1 and 6 staining than the corresponding normal breast tissues, but ELOVL5 was not stained strongly in the TN or EP+H- tumors. Furthermore, the TN tumors exhibited stronger ELOVL1 and 6 staining than the EP+H- tumors.

**Conclusions:**

Marked differences in fatty acid metabolism pathways, including those related to ELOVL1 and 6, were detected between TN and EP+H-, and it was suggested that ELOVL1 and 6-related fatty acid metabolism pathways may be targets for therapies against TN.

**Electronic supplementary material:**

The online version of this article (doi:10.1186/s12885-017-3554-4) contains supplementary material, which is available to authorized users.

## Background

Of all the types of cancer affecting women, breast cancer exhibits the highest morbidity rate [1]. World Cancer Research Fund International reported that nearly 1.7 million new breast cancer cases are diagnosed annually worldwide, and Center for Cancer Control and Information Service of National Cancer Center Japan released that nearly 74,000 new breast cancer cases are diagnosed annually in Japan. Breast cancer is a heterogeneous form of cancer with various biological characteristics, and it is classified into various clinical subtypes based on the presence or absence of the estrogen receptor (ER), progesterone receptor (PgR), and human epidermal growth factor receptor-2 (HER2). The treatment varies according to the subtype [2]. Recent therapeutic advances have included molecular targeted treatment [3]. Most breast cancer subtypes are ER-positive [4], but approximately 15–20% do not express ER, PgR, or HER2. These are known as triple-negative breast cancer (TN). TN is associated with a high recurrence rate, distant metastasis, and a poor survival. It is the most aggressive breast cancer [5, 6]. The prevalence of TN is the highest in premenopausal African American women, and a recent report notes that 39% of all African American premenopausal women diagnosed with breast cancer are diagnosed with TN. The prevalence of TN in non-African American women of the same age is much less, approximately 15 to 20%. [2, 5]. Anticancer drug therapy is the only effective systemic treatment for TN [7]. Therefore, it is necessary to understand the characteristics of TN to aid the development of effective systemic treatments for the disease.

In research into cancer biology, metabolome profiling is important for finding central metabolic changes. Cancer cells act differently and have the different microenvironments in comparison to normal cells. Therefore, cancer cells acquire the ability to adapt to special environments including hypoxic conditions. For example, cancer cells generate ATP from glycolysis by suppressing ATP production from oxidative phosphorylation, which is a phenomenon well-known as the “Warburg effect” [8–10]. Additionally, global reprogramming occurs in amino acid metabolism [11]. Therefore, a bigger picture of cancer metabolism can be evaluated by linking with glycolysis and amino acid metabolism, and understandings of these metabolic changes may lead to new cancer strategies, so it is important to study cancer metabolism. The metabolome helps characterize the phenotype of cells and tissues, potentially shedding new light on cell functions and biological changes [12–14]. In the past 10 years, metabolome analysis, which involves the analysis of metabolite levels in the body, has developed rapidly in various research fields, such as clinical research, cell biology, and plant/food science [15–18]. Understanding cell activity has benefited from analysis of the genome (DNA), transcriptome (RNA), and proteome (protein). However, in addition to these large molecules, low-molecular-weight molecules, such as amino acids, organic acids, and fatty acids, are abundant in the body. To more fully understand global cellular activity, these low-molecular-weight molecules should be also analysed.

Recently, metabolome analysis of breast cancer has also started to be performed. For example, Pelicano et al. identified differences in glycolysis metabolism between TN and other breast cancer subtypes. They suggested that the glycolytic inhibitor was effective against TN [19]. Guo et al. investigated de novo lipogenesis in tissue samples from 134 patients with six types of cancer (breast, lung, colorectal, esophageal, gastric and thyroid). The changes that they found in the degree of lipid unsaturation generated by lipogenic enzymes in the cancer microenvironment may have implications for understanding carcinogenesis [20]. Budczies and colleagues analyzed glutamine metabolism in breast cancer [21], and potential new cancer treatments against TN, such as glutaminase inhibitors, are being considered [22]. In addition, various studies for the comparison of metabolome between breast cancer subtypes have been also performed [23–25].

Methods commonly used for metabolome analysis include liquid chromatography/mass spectrometry (LC/MS), gas chromatography/mass spectrometry, nuclear magnetic resonance, and capillary electrophoresis mass spectrometry. However, hydrophobic and hydrophilic metabolites can be comprehensively and sensitively analyzed by using LC/MS [26]. In this study, we analyzed the metabolomes of 74 breast cancer tissue samples paired with the normal breast tissue samples using LC/MS. Furthermore, we evaluated differences in the metabolome between TN and breast cancer tissue samples that were ER- and PgR-positive, but HER2-negative (designated as EP + H-). Because of the biochemical characteristics of breast cancer differ according to the subtype, and its metabolic profile also seems to vary according to the subtype [23, 27]. In these experiments, we found differences in the profiles of very long-chain fatty acids, indicating changes in the metabolic pathway according to breast cancer subtypes. These results may inform the development of novel treatment against TN.

## Methods

### Sample collection

This study was approved by the ethics committee at Kobe University Graduate School of Medicine (Kobe, Japan) and was conducted between October 2013 and November 2015. Human tissue samples were used in accordance with the guidelines of Kobe University Hospital, and written informed consent was obtained from all subjects. The tissue samples were collected from the patients with diagnosis of invasive breast cancer and with its surgical operation at Kobe University Hospital and Hyogo Cancer Center, and the male patients, the patients under 18 years old, and the patients who had a history of cancer before being diagnosed were excluded. The tissue samples were collected prior to the beginning of adjuvant therapy. After surgery, the breast cancer and normal breast tissue samples were immediately cut into pieces. The normal breast tissue samples were obtained from sites that were a sufficient distance from the cancer tissue sampling sites. Breasts resected by surgery were pathologically diagnosed, and it was confirmed whether the sites of resected tissue samples were cancer or normal breast. We defined EP + H- as follow: ER and PgR was more than 3 (total score of Allred score) or more than 3a (J-score). Regarding HER2, the immunohistochemistry test was 0 and 1+. If the immunohistochemistry test was 2+, fluorescence in the situ hybridization (FISH) test was less than 2.2. Pathologically, all of the primary tumors measured <5 cm in diameter (T1 and T2 which is defined by TNM classification of the Unio Internationalis Contra Cancrum). The tissue samples were transferred to clean tubes containing dry ice and kept in a deep freezer at −80 °C until use. For the experiments, tissue samples were defrosted on ice and cut into pieces of about 5 mg each. The total number of breast cancer tissue samples was 74, and the numbers of EP + H- and TN were 49 and 11, respectively. Regarding the remaining 14 samples, the subtype with ER-, PgR+ and HER2- was 3 samples, and the subtype with ER+, PgR- and HER2- was 8 samples, the subtype with and ER+, PgR+ and HER2+ was 3 samples.

### Sample preparation for the analysis of hydrophilic compounds

Each 5 mg sample was homogenized using an Automill TK-AM5 (Tokken, Inc., Chiba, Japan) with 0.9 mL of a solvent mixture (MeOH, H_2_O, and CHCl_3_ in a ratio of 2.5:1:1) containing 1 μM 2-bromohypoxanthine and 1 μM 10-camphorsulfonic acid as internal standards, and then low-molecular-weight metabolites were extracted as previously described [15]. The extracted solutions were subjected to LC/MS analysis.

### Sample preparation for lipid compounds

For lipid compound analysis, 5 mg of tissue was homogenized with 225 μL of MeOH and 25 μL of 500 μg/L dilauroylphosphatidylcholine (PC 12:0/12:0; Avanti Polar Lipids, AL, USA) dissolved in MeOH as the internal standard. After being left on ice, the solution was centrifuged at 16,000×g for 5 min at 4 °C, and The extracted solutions were subjected to LC/MS analysis.

### Liquid chromatography/mass spectrometry

According to the method previously described [15, 28], LC/MS was carried out using a Nexera LC system (Shimadzu Co., Kyoto, Japan) equipped with two LC-30 AD pumps, a DGU-20As degasser, a SIL-30 AC autosampler, a CTO-20 AC column oven and a CBM-20A control module, coupled with an LCMS-8040 triple quadrupole mass spectrometer (Shimadzu Co.).

### Data analysis

To identify the hydrophilic cationic and anionic metabolites, the m/z value and retention time of each peak were compared with those of chemical standards that had been analysed using the same methods [15, 28]. Peak picking and integration were automatically performed using the LabSolutions software (ver. 5.65; Shimadzu Corp.), and the results were then checked manually. The peak area of each metabolite was normalized to that of the internal standards. Regarding the hydrophobic lipid metabolites, analysis based upon physicochemical properties and/or spectral similarity with public or commercial spectral libraries was performed (putative annotation), because chemical reference standards could not be obtained. Mouse liver extract was used as the quality control sample to ensure consistency in retention time information in the in-house library and detected peak retention time during the experiment. A quality control sample was included in each analyzing batch for the hydrophobic lipid metabolites. A blank sample was also used for each analysis. When samples were prepared for hydrophilic and hydrophobic analysis, we utilized blank samples that did not contain patient samples. A blank sample was included in each analyzing batch. In the analysis of blank samples, we confirmed that there is no peak detection in the blank samples. We also checked that there is no error from the detection value of the internal standards during the measurements. Numbers of targeted metabolites in multiple reaction monitoring (MRM)-based database were 267 of hydrophilic metabolites and 284 of hydrophobic lipid metabolites (Additional file 1: Table S1, Additional file 2: Table S2 and Additional file 3: Table S3). The metabolites with the lower intensity near the detection limit were excluded from the evaluations, because the lower intensity might be accurate data.

### Real-time reverse transcription polymerase chain reaction (RT-PCR)

RNA was extracted from the human tissue samples using the NucleoSpin RNA® kit (TaKaRa Bio Inc., Tokyo, Japan) according to the manufacturer’s procedure. We performed RNA extraction using 8 TN samples, 8 EP + H- samples, and the corresponding normal breast tissue samples because there were only 8 TN tumors remaining. Then, cDNA was produced from the collected RNA with the RT^2^ first strand kit (Qiagen, Valencia, CA, USA) according to the manufacturer’s procedure. The quantitative real-time RT-PCR was conducted using the 7500 real time PCR system (Applied Biosystems, Tokyo Japan) and Power SYBR Green PCR master mix (Applied Biosystems). The expression levels of the target mRNA were calculated using the threshold cycle (Ct) value of the relevant PCR product, and were normalized to the mRNA expression level of 18S rRNA. All mRNA expression levels of fatty chain elongases (elongation of very long chain fatty acids [ELOVL]) 1–7 were calculated by using the comparative 2ΔΔCt method. The primer sequences used in this experiment are as follows: ELOVL1_F AGCACATGACAGCCATTCAG and ELOVL1_R AGATGGTGCCATACATCCAG; ELOVL2_F TGCTCTTTCTCTCAGGGGTATC and ELOVL2_R AGTTGTAGCCTCCTTCCCAAG; ELOVL3_F TTCGAGGAGTATTGGGCAAC and ELOVL3_R GAAGATTGCAAGGCAGAAGG; ELOVL4_F TGGTGGAAACGATACCTGAC and ELOVL4_R AATTAGAGCCCAGTGCATCC; ELOVL5_F CATTCCCTCTTGGTTGGTTG and ELOVL5_R TTCAGGTGGTCTTTCCTTCG; ELOVL6_F GGATGCAGGAAAACTGGAAG and ELOVL6_R ATTCATTAGGTGCCGACCAC; ELOVL7_F GCGCAAGAAAAATAGCCAAG and ELOVL7_R GAATGTTCCCAAACCACCTG; 18S rRNA_F AAACGGCTACCACATCCAAG and 18S rRNA_R CCTCCAATGGATCCTCGTTA.

### Immunohistochemistry

We subjected sections of the TN tumors, EP + H- tumors, and the corresponding normal breast tissue samples to immunohistochemistry. From each group, we stained the 5 tissue sections for ELOVL proteins and the 4 sections for choline kinase. To match characteristics of tumor, we selected these sections. All sections had the similar characteristics pathologically as the primary tumor (histologic grade, tumor size, lymph node involevement and stage). The sections were cut from formalin-fixed and paraffin-embedded tissues. The paraffin sections were heated in an incubator at 65 °C for 30 min and then deparaffinized with xylene and rehydrated in 100% ethanol, 90% ethanol, 70% ethanol and phosphate-buffered saline. The sections were placed in a Decloaking Chamber (Biocare Medical, Concord, CA, USA) with Target Retrieval Solution (Dako, Tokyo, Japan) at 125 °C for 3 min, at 90 °C for 10 s, and then left at room temperature for 20 min. Endogenous peroxidase was inactivated using Peroxidase-Blocking Solution (Dako, Tokyo, Japan). The sections were incubated with a primary antibody against choline kinase at a dilution of 1:20, a primary antibody against ELOVL1 at a dilution of 1:40, primary antibody against ELOVL5 at a dilution of 1:50, and a primary antibody against ELOVL6 at a dilution of 1: 200 in a humidified chamber at 4 °C overnight. The sections were washed by using Tris-buffered saline with Tween 20 and then incubated with EnVision + System- HRP Labelled Polymer Anti-Rabbit (Dako, Tokyo, Japan) as a secondary antibody against rabbit IgG (Dako, Tokyo, Japan) for 30 min at room temperature. Next, the sections were stained with ImmPACT DAB (Vector, Tokyo, Japan) for 2–3 min and then washed with water. Subsequently, nuclei in the sections were counterstained with Mayer’s hematoxylin for 3 min, and then the sections were dehydrated with 70% ethanol, 80% ethanol, 90% ethanol and 100% ethanol, and were finally cleared by xylene.

### Hematoxylin & eosin (HE) staining

Sections were cut from formalin-fixed and paraffin-embedded tissues. The paraffin sections were heated in an incubator at 65 °C for 30 min, then deparaffinized by xylene and rehydrated in 100% ethanol, 90% ethanol, 70% ethanol and phosphate buffered saline. Then, the sections were stained with Mayer’s hematoxylin for 10 min (to stain the nuclei) and washed with water, before being stained with eosin for 10 min (to stain the cytoplasm). The stained sections were dehydrated in 70% ethanol, 80% ethanol, 90% ethanol, 100% ethanol and finally cleared by xylene.

### Statistical analyses

The Mann-Whitney U test was used for comparisons of median age and tumor size. The chi-squared test was used for comparisons of histological type, histological grade, lymph node involvement, and cancer stage. The levels of tissue metabolites were compared between pairs of breast cancer tissue samples and normal breast tissue samples using the Wilcoxon signed rank test. The Mann-Whitney U test was used for comparisons involving the metabolites that were not detected in the paired samples. The levels of tissue metabolites were compared between the TN and EP + H- tumors using the Mann-Whitney U test. Furthermore, the levels of tissue metabolites were compared between pairs of TN/ EP + H- tumors and the corresponding normal breast tissue samples using the Wilcoxon signed rank test. The Mann-Whitney U test was used for comparisons involving the metabolites that were not detected in the paired samples. The mRNA expression levels of ELOVL1, 2, 3, 4, 5, 6, and 7, which were determined by RT-PCR, was compared between TN tumors and the corresponding normal tissues and between EP + H- tumors and the corresponding normal tissues using the Wilcoxon signed rank test. The Mann-Whitney U test was used for comparisons TN tumors and HR tumors. In addition, to investigate whether the metabolite changes were real and biological, and could be false positives/random, false discovery rate (FDR)-adjusted p- values were calculated. In all cases, *p*-values of <0.05 were considered to indicate a significant difference. All analyses were performed using the default conditions of JMP13 (SAS Institute, Inc.).

## Results

### Comparisons between the breast cancer and normal breast tissue samples

We analyzed the metabolites in the 74 breast cancer tissue samples and the corresponding normal breast tissue samples using LC/MS. The subjects’ characteristics are shown in Table 1. In this study, we performed the MRM-based targeted analysis. The number of targeted metabolites included in the MRM database was 267 of hydrophilic metabolites and 284 of hydrophobic lipid metabolites (Additional file 1: Table S1, Additional file 2: Table S2 and Additional file 3: Table S3), and we identified 142 of hydrophilic metabolites and 278 of hydrophobic lipid metabolites in breast cancer tissue samples and the corresponding normal breast tissue samples. Levels of the cationic metabolites (such as nucleobases and derivatives, nucleosides, amino acids and derivatives, quaternary ammonium salts and folic acids), anionic metabolites (such as organic acids, fatty acids, benzoic acids, sugar phosphates, coenzyme A and nucleosides) and lipid metabolites (such as lyso-glycerophosphocholines [LPC], glycerophosphocholines [PC], lyso-glycerophosphoethanolamines [PE], glycerophosphoethanolamines [LPE], free fatty acids and cholic acids) are shown in Additional file 4: Table S4 and Additional file 5: Table S5.

**Table 1:** Characteristics of the study subjects

	Breast cancer	TN	EP + H-	*p* value
Number of patients	74	11	49	
Age median	Median: 64.6 Max: 89 Min: 37	Median: 69.5 Max: 77 Min: 62	Median: 62.8 Max: 89 Min: 38	0.0781
Histologic type				
Invasive ductal carcinoma	64	8	45	0.0744
Other	10	3	4	
Histologic grade				
grade 1	37	3	28	0.0623
grade 2	15	2	11	
grade 3	22	6	10	
Tumor size (cm)	Median: 2.3 Max: 5.0 Min: 0.2	Median: 2.1 Max: 5.0 Min: 0.9	Median: 2.4 Max: 5.0 Min: 0.5	0.2521
Lymph node involvement				
Positive	24	1	18	0.0749
Negative	50	10	31	
pStage				
I	27	5	16	0.068
IIa	27	6	18	
IIb	14	0	11	
IIIa	5	0	4	
IIIc	1	0	0	

Tissues from 74 patients with breast cancer were studied, and included the samples of the tumour and corresponding normal breast tissue samples from each patient. Tumors were characterized as TN (*n* = 11) or EP + H- (*n* = 49). The *p*-values were calculated during comparisons between the TN and EP + H- tumors. The Mann-Whitney U-test was used for comparisons of the median age and tumor size. The chi-square test was used for comparisons of histological type, histological grade, lymph node involvement, and disease stage

**Fig. 1:**
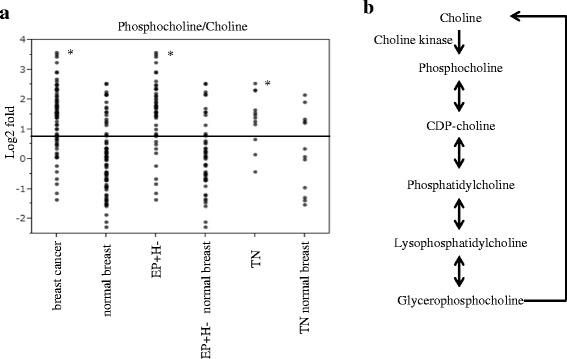
Ratios of phosphocholine to choline in breast cancer and corresponding normal breast tissue samples. **a** A dot plot of the ratio of phosphocholine to choline in all breast cancer tissue samples, TN tumors, EP + H- tumors, and the corresponding normal breast tissue samples. Asterisks indicate significant differences (*p* < 0.05) between the breast cancer tissue samples, TN tumors, EP + H- tumors, and the corresponding normal breast tissue samples. The Wilcoxon signed rank test was used for comparisons between each type of tissue. The Mann-Whitney U-test was used for comparisons between TN and EP + H-. The vertical axis shows the log2 fold values of the phosphocholine to choline ratio. In the x-axis, EP + H- normal breast and TN normal breast indicate the normal breast tissue samples corresponding to each cancer subtype. **b** The choline pathway including choline kinase

**Fig. 2:**
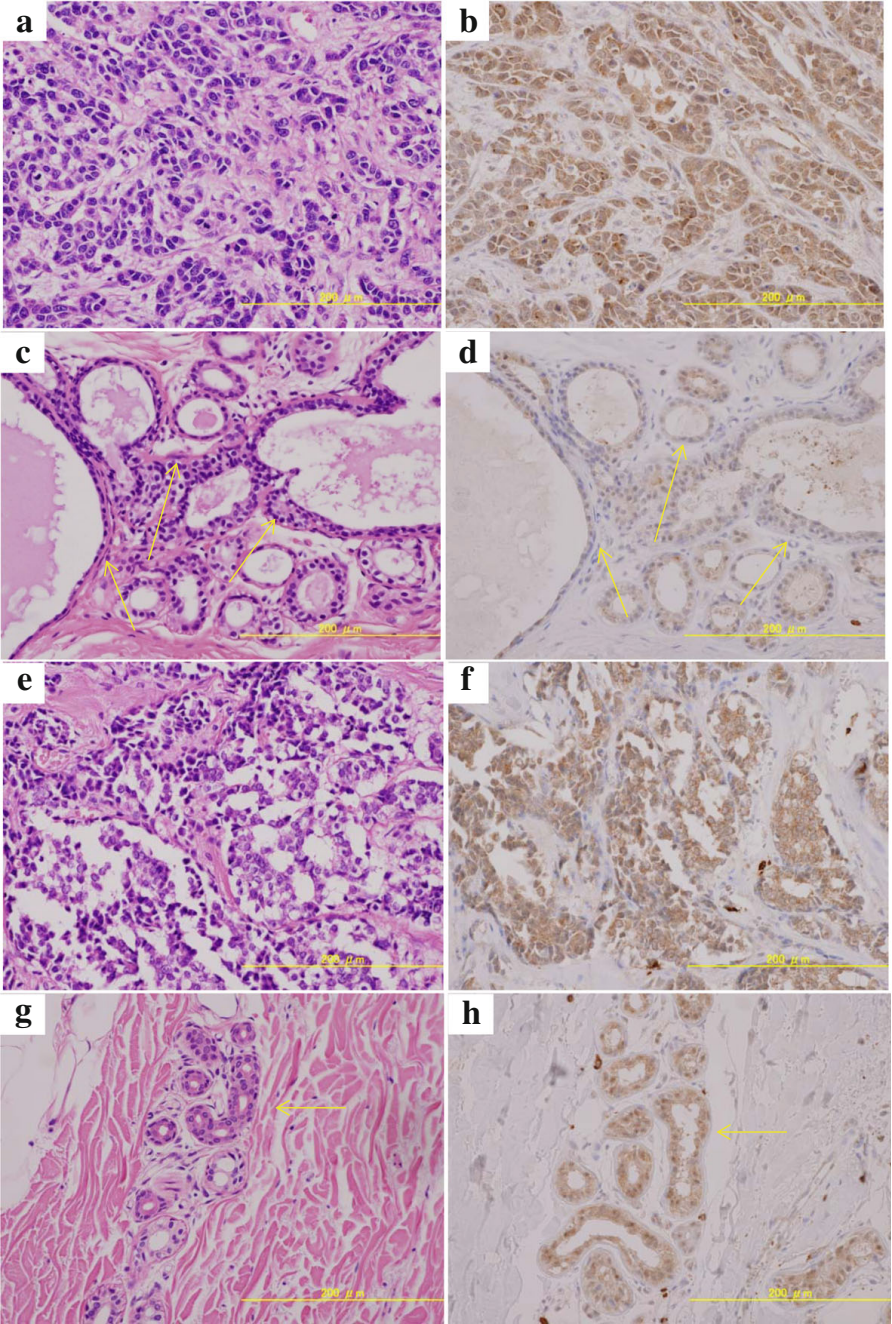
Immunostaining and HE staining of choline kinase in breast cancer and corresponding normal breast tissues. **a** and **b** HE staining and immunostaining of choline kinase in the TN tumors; **c** and **d** HE staining and immunostaining of choline kinase in the normal breast tissue samples corresponding to the TN tumors; **e** and **f** HE staining and immunostaining of choline kinase in the EP + H- tumors; **g** and **h** HE staining and immunostaining of choline kinase in the normal breast tissue samples corresponding to the EP + H- tumors. The arrows show the normal breast tissue samples

**Fig. 3:**
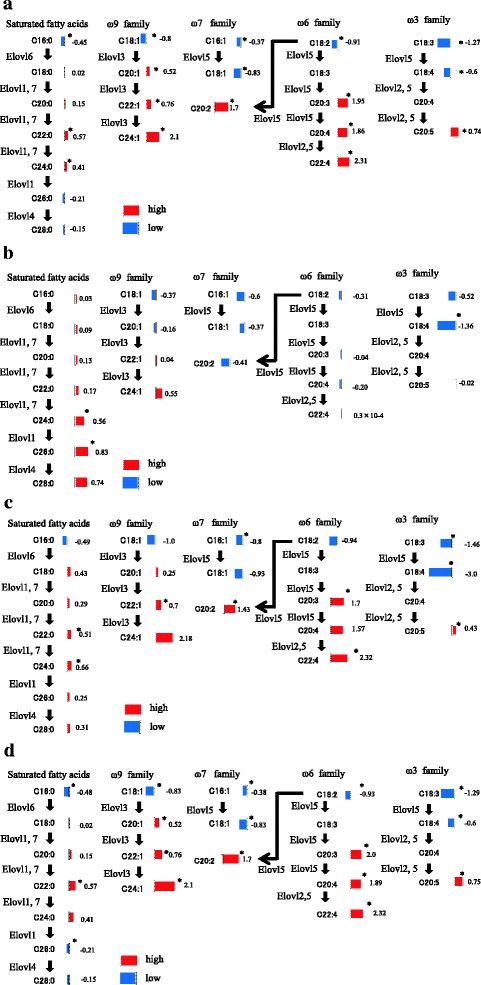
Fatty acid levels and related metabolic pathways in breast cancer and corresponding normal breast tissue. **a** Comparison between the breast cancer tissue samples and the corresponding normal breast tissue samples (red bars: cancer > normal; blue bars: cancer < normal); **b** Comparison between the TN tumors and EP + H- tumors (red bars: TN > EP + H-; blue bars: TN < EP + H-); **c** Comparison between the TN tumors and the corresponding normal breast tissue samples (red bars: cancer > normal; blue bars: cancer < normal); **d** Comparison between the EP + H- tumors and the corresponding normal breast tissue samples (red bars: cancer > normal; blue bars: cancer < normal). Asterisks indicate significant differences (*p* < 0.05). The Wilcoxon signed rank test was used for comparisons between the pairs of breast cancer tissue samples and normal breast tissue samples, and the Mann-Whitney-U test was used for comparisons involving metabolites that were not detected in the paired samples. The scale of each bar reflects the ratio of the log2 fold values of the pairs of breast cancer tissue samples and the corresponding normal breast tissue samples. The numbers next to each bar indicate the Log2-fold values (**a** the breast cancer tissue samples versus the corresponding normal breast tissue samples; **b** TN tumors versus EP + H- tumors; **c** TN tumors versus the corresponding normal breast tissue samples; **d** EP + H- tumors versus the corresponding normal breast tissue samples)

Next, we confirmed the association between the identified metabolites and metabolite pathways by using the Kyoto Encyclopedia of Genes and Genomes (KEGG) Database, and evaluations based on the glycolytic pathway, tricarboxylic acid (TCA) cycle, glutamine pathway, choline pathway, urea cycle, tryptophan cycle, glutathione cycle, purine pathway, pyrimidine pathway, and amino acid metabolism were carried out (Table 2A). In the analysis of the levels of metabolites related to the glycolytic pathway, the level of lactic acid was significantly increased between breast cancer tissue samples and the corresponding normal tissue samples, but no significant differences in other metabolites were detected. In the TCA cycle, the levels of cis-aconitic acid and isocitric acid were significantly higher, and those of 2-ketoglutaric acid and succinic acid were significantly lower in the breast cancer tissues than in normal tissue. Regarding the levels of metabolites related to the glutamine pathway, the breast cancer tissue samples displayed higher levels of glutamine and glutamic acid than the normal breast tissue samples. In the analysis of the metabolites related to the choline pathway, the breast cancer tissue samples demonstrated significantly higher levels of choline and phosphocholine than the normal breast tissue samples, and the ratio of phosphocholine to choline was higher in the breast cancer tissue samples. Figure 1 shows the levels of metabolites related to the choline pathway and the phosphocholine to choline ratio in the breast cancer tissue samples and the corresponding normal breast tissue samples. During immunostaining, it was demonstrated that the expression of choline kinase, which is an enzyme that catalyzes the conversion of choline to phosphocholine, was upregulated in the breast cancer tissue samples compared with the normal breast tissue samples (Fig. 2). In the analysis of the metabolites related to the urea cycle, the levels of aspartate, arginine and citrulline were significantly higher in breast cancer tissue samples. Regarding the metabolites related to the purine pathway, the levels of guanosine and hypoxanthine were higher in the breast cancer tissue samples. Most amino acids exhibited significantly higher levels in the breast cancer tissue samples. As for saturated fatty acids, the breast cancer tissue samples exhibited lower levels of C16:0 than the normal breast tissue samples, and the levels of C20:0, C22:0, and C24:0 were higher in the breast cancer tissue samples. Furthermore, the levels of C26:0 and C28:0 were lower in the breast cancer tissue samples, although C26:0 and C28:0 showed no significant changes in FDR-adjusted *p*-value. Concerning unsaturated fatty acids, the levels of ω3 family C18:3 and C18:4 were lower and of C20:5 higher in the breast cancer tissues. The level of the ω6 family C18:2 was lower, and the levels of C20:3, C20:4 and C22:4 were significantly higher in the breast cancer tissues. The levels of the ω7 family C16:1 and C18:1 were significantly lower in the cancer tissues, although C16:1 showed no significant changes in FDR-adjusted *p*-value. In the ω9 family, C18:1 was lower, but the levels of C20:1, C22:1 and C24:1 were significantly higher (Fig. 3a). The levels of most LPC, LPE, PC, and PE were higher in the breast cancer tissue samples than in the normal breast tissue samples, and a summary of our findings regarding glycerophospholipids is shown in Table 3.

**Table 2:** Evaluation of metabolite pathways in breast cancer and the corresponding normal breast tissue samples

A				
Pathway	Biochemical name	Log2-fold (Breast cancer/Normal breast)	*p*-value	FDR-adjusted *p*-value
Glycolysis	G1P (glucose-1-phosphate)	−0.280	0.261	0.315
	G6P (Glucose-6-phosphate)	−0.370	0.065	0.076
	F6P (Fructose-6-phosphate)	−0.071	0.837	0.809
	FBP (fructose-1,6-bisphosphate)	0.190	0.470	0.688
	GAP (Glyceraldehyde-3-phosphate)	−0.261	0.163	0.327
	PEP (Phosphoenol-pyruvate)	0.087	0.925	0.829
	Pyruvic acid	−0.058	0.487	0.788
	Lactic acid	1.828	<0.0001	<0.0001
TCA cycle	Pyruvic acid	−0.058	0.487	0.788
	Lactic acid	1.828	<0.0001	<0.0001
	Acetyl-CoA	0.043	0.921	0.902
	Oxaloacetic acid	−0.364	0.134	0.031
	Citric acid	−0.009	0.307	0.962
	cis-Aconitic acid	1.028	<0.0001	<0.0001
	Isocitric acid	0.382	0.016	0.036
	2-Ketoglutaric acid	−0.791	<0.0001	<0.0001
	Succinic acid	−0.954	<0.0001	<0.0001
	Fumaric acid	0.071	0.617	0.789
	Malic acid	0.103	0.436	0.660
Glutamine pathway	L-Glutamate	1.404	<0.0001	<0.0001
	L-Glutamine	1.650	<0.0001	<0.0001
	GABA	0.109	0.890	0.696
Choline pathway	Choline	0.913	<0.0001	<0.0001
	Phosphocholine	2.784	<0.0001	<0.0001
	Betaine	2.256	<0.0001	<0.0001
Urea cycle	L-Asparate	0.636	<0.0001	<0.0001
	Fumaric acid	0.071	0.617	0.787
	L-Arginine	1.314	<0.0001	<0.0001
	L-Ornithine	−0.120	0.766	0.660
	L-Citrulline	0.925	<0.0001	<0.0001
	Uric Acid	−0.073	0.185	0.667
Tryptophan cycle	L-Tryptophan	−0.792	<0.0001	<0.0001
	L-Kynurenine	−0.079	0.404	0.754
	Anthranilic acid	−0.313	0.291	0.372
Glutathione cycle	GSH	0.732	0.012	0.017
	GSSG	−0.148	0.505	0.705
	gamma-L-Glutamylcysteine	0.084	0.800	0.799
	Glycine	2.388	<0.0001	<0.0001
	L-Cysteine	0.271	0.065	0.150
Purine pathway	Guanine	0.093	0.355	0.688
	Guanosine	1.449	<0.0001	<0.0001
	Hypoxanthine	1.448	<0.0001	<0.0001
	Xanthine	0.165	0.315	0.447
	Adenine	−0.027	0.837	0.9480
	Adenosine	0.301	0.035	0.200
Pyrimidine pathway	Cytidine	−0.073	0.660	0.787
	Cytosine + Histamine	−0.213	0.212	0.269
	beta-Alanine	1.664	<0.0001	<0.0001
	Thymine	0.043	0.908	0.910
	Uridine	0.926	<0.0001	<0.0001
Amino acid metabolism	Glycine	2.388	<0.0001	<0.0001
	L-Arginine	1.314	<0.0001	<0.0001
	L-Asparate	0.636	<0.0001	<0.0001
	L-Asparagine	0.524	<0.0001	0.0025
	L-Cysteine	0.271	0.065	0.150
	L-Cystine	−0.260	0.217	0.338
	L-Lysine	1.203	<0.0001	<0.0001
	L-Glutamine	1.650	<0.0001	<0.0001
	L-Glutamate	1.404	<0.0001	<0.0001
	L-Histidine	0.719	<0.0001	0.00080
	L-Isoleucine	1.597	<0.0001	<0.0001
	L-Leucine	1.386	<0.0001	<0.0001
	L-Methionine	1.016	<0.0001	<0.0001
	L-Phenylalanine	1.493	<0.0001	<0.0001
	L-Proline	1.714	<0.0001	<0.0001
	L-Serine	1.484	<0.0001	<0.0001
	L-Threonine	1.261	<0.0001	<0.0001
	L-Tryptophan	−0.792	<0.0001	<0.0001
	L-Tyrosine	1.271	<0.0001	<0.0001
	L-Valine	1.267	<0.0001	<0.0001
B				
Pathway	Biochemical name	Log2-fold (TN/EP + H-)	*p*-value	FDR-adjusted *p*-value
Glycolysis	G1P (glucose-1-phosphate)	0.463	0.092	0.689
	G6P (Glucose-6-phosphate)	0.090	0.685	0.931
	F6P (Fructose-6-phosphate)	0.821	0.005	0.040
	FBP (fructose-1,6-bisphosphate)	−0.665	0.330	0.689
	GAP (Glyceraldehyde-3-phosphate)	0.641	0.036	0.511
	PEP (Phosphoenol-pyruvate)	0.743	0.095	0.643
	Pyruvic acid	−0.148	0.555	0.906
	Lactic acid	0.302	0.272	0.689
TCA cycle	Pyruvic acid	−0.148	0.555	0.906
	Lactic acid	0.302	0.272	0.689
	Acetyl-CoA	−0.140	0.883	0.931
	Oxaloacetic acid	0.461	0.077	0.511
	Citric acid	−0.272	0.420	0.770
	cis-Aconitic acid	0.200	0.474	0.781
	Isocitric acid	−0.058	0.598	0.937
	2-Ketoglutaric acid	0.155	0.657	0.906
	Succinic acid	−0.825	0.011	0.511
	Fumaric acid	−0.277	0.419	0.707
	Malic acid	0.330	0.228	0.689
Glutamine pathway	L-Glutamate	0.557	0.005	0.045
	L-Glutamine	−0.281	0.242	0.689
	GABA	0.483	0.007	0.689
Choline pathway	Choline	0.491	0.041	0.511
	Phosphocholine	0.032	0.779	0.937
	Betaine	−0.448	0.978	0.835
Urea cycle	L-Asparate	0.091	0.697	0.931
	Fumaric acid	−0.277	0.419	0.707
	L-Arginine	−0.301	0.399	0.770
	L-Ornithine	−0.009	0.906	0.979
	L-Citrulline	0.243	0.130	0.906
	Uric Acid	0.221	0.305	0.906
Tryptophan cycle	L-Tryptophan	0.182	0.182	0.906
	L-Kynurenine	0.377	0.157	0.689
	Anthranilic acid	−1.492	0.020	0.066
Glutathione cycle	GSH	−0.488	0.291	0.770
	GSSG	0.289	0.304	0.718
	gamma-L-Glutamylcysteine	−0.261	0.254	0.835
	Glycine	0.333	0.144	0.689
	L-Cysteine	0.379	0.102	0.689
Purine pathway	Guanine	−0.075	0.935	0.931
	Guanosine	−0.130	0.683	0.906
	Hypoxanthine	0.277	0.201	0.689
	Xanthine	0.046	0.978	0.937
	Adenine	−0.901	0.467	0.740
	Adenosine	−0.570	0.214	0.689
Pyrimidine pathway	Cytidine	−0.268	0.710	0.835
	Cytosine + Histamine	0.212	0.493	0.835
	beta-Alanine	0.078	0.618	0.931
	Thymine	−0.842	0.310	0.689
	Uridine	0.111	0.697	0.906
Amino acid metabolism	Glycine	0.333	0.144	0.689
	L-Arginine	−0.301	0.399	0.770
	L-Asparate	0.091	0.697	0.931
	L-Asparagine	0.008	0.849	0.979
	L-Cysteine	0.379	0.102	0.689
	L-Cystine	0.160	0.775	0.927
	L-Lysine	−0.058	0.964	0.937
	L-Glutamine	−0.281	0.242	0.689
	L-Glutamate	0.557	0.005	0.045
	L-Histidine	0.426	0.235	0.689
	L-Isoleucine	0.172	0.177	0.931
	L-Leucine	0.047	0.389	0.937
	L-Methionine	0.128	0.257	0.931
	L-Phenylalanine	0.046	0.508	0.937
	L-Proline	0.085	0.474	0.937
	L-Serine	−0.042	0.935	0.937
	L-Threonine	0.304	0.441	0.689
	L-Tryptophan	0.182	0.182	0.906
	L-Tyrosine	0.055	0.474	0.937
	L-Valine	0.269	0.071	0.770
C				
Pathway	Biochemical name	Log2-fold (TN/Normal breast)	*p*-value	FDR-adjusted *p*-value
Glycolysis	G1P (glucose-1-phosphate)	−0.428	0.594	0.510
	G6P (Glucose-6-phosphate)	−0.230	0.699	0.665
	F6P (Fructose-6-phosphate)	0.394	0.357	0.422
	FBP (fructose-1,6-bisphosphate)	−0.606	0.835	0.607
	GAP (Glyceraldehyde-3-phosphate)	−0.006	0.939	0.990
	PEP (Phosphoenol-pyruvate)	1.241	0.030	0.072
	Pyruvic acid	−0.231	0.341	0.656
	Lactic acid	2.501	<0.0001	<0.0001
TCA cycle	Pyruvic acid	−0.231	0.341	0.656
	Lactic acid	2.501	<0.0001	0.001
	Acetyl-CoA	0.361	0.516	0.646
	Oxaloacetic acid	0.112	0.840	0.821
	Citric acid	−0.104	0.665	0.856
	cis-Aconitic acid	1.428	0.003	0.0060
	Isocitric acid	0.669	0.479	0.248
	2-Ketoglutaric acid	−0.760	0.112	0.076
	Succinic acid	−1.624	0.003	0.0017
	Fumaric acid	−0.078	0.921	0.872
	Malic acid	0.570	0.132	0.263
Glutamine pathway	L-Glutamate	1.673	<0.0001	<0.0001
	L-Glutamine	1.631	<0.0001	<0.0001
	GABA	0.321	0.371	0.402
choline pathway	Choline	1.493	0.002	0.0073
	Phosphocholine	2.442	<0.0001	<0.0001
	Betaine	2.357	<0.0001	0.011
Urea cycle	L-Asparate	0.587	0.175	0.318
	Fumaric acid	−0.078	0.921	0.872
	L-Arginine	0.917	0.006	0.027
	L-Ornithine	−0.515	0.471	0.340
	L-Citrulline	1.217	<0.0001	0.010
	Uric Acid	−0.016	0.665	0.656
Tryptophan cycle	L-Tryptophan	−0.785	0.040	0.045
	L-Kynurenine	0.194	0.559	0.713
	Anthranilic acid	−1.739	0.112	0.130
Glutathione cycle	GSH	0.969	0.143	0.263
	GSSG	−0.443	0.188	0.327
	gamma-L-Glutamylcysteine	−0.436	0.270	0.646
	Glycine	2.790	<0.0001	<0.0001
	L-Cysteine	0.621	0.046	0.063
purine pathway	Guanine	0.150	0.689	0.745
	Guanosine	1.680	<0.0001	<0.0001
	Hypoxanthine	2.389	<0.0001	<0.0001
	Xanthine	0.079	0.708	0.872
	Adenine	−0.229	0.663	0.844
	Adenosine	−0.125	0.689	0.826
Pyrimidine pathway	Cytidine	−0.567	0.215	0.263
	Cytosine + Histamine	−0.343	0.373	0.452
	beta-Alanine	1.814	<0.0001	<0.0001
	Thymine	−0.426	0.897	0.665
	Uridine	1.072	0.012	0.028
Amino acid metabolism	Glycine	2.790	<0.0001	<0.0001
	L-Arginine	0.917	0.006	0.027
	L-Asparate	0.587	0.175	0.318
	L-Asparagine	0.809	0.035	0.064
	L-Cysteine	0.621	0.046	0.063
	L-Cystine	−0.039	0.840	0.235
	L-Lysine	1.094	0.012	0.021
	L-Glutamine	1.631	<0.0001	<0.0001
	L-Glutamate	1.673	<0.0001	<0.0001
	L-Histidine	1.056	0.040	0.084
	L-Isoleucine	1.723	<0.0001	0.00016
	L-Leucine	1.479	0.003	0.0073
	L-Methionine	1.219	0.006	0.0030
	L-Phenylalanine	1.426	0.002	0.0099
	L-Proline	2.017	0.002	0.0012
	L-Serine	1.474	0.002	0.0079
	L-Threonine	1.492	0.0011	0.0073
	L-Tryptophan	−0.785	0.040	0.035
	L-Tyrosine	1.383	<0.0001	0.00026
	L-Valine	1.458	<0.0001	0.00015
D				
Pathway	Biochemical name	Log2-fold (EP + H- /Normal breast)	*p*-value	FDR-adjusted *p*-value
Glycolysis	G1P (glucose-1-phosphate)	−0.222	0.422	0.559
	G6P (Glucose-6-phosphate)	−0.404	0.131	0.083
	F6P (Fructose-6-phosphate)	−0.440	0.185	0.068
	FBP (fructose-1,6-bisphosphate)	0.345	0.525	0.548
	GAP (Glyceraldehyde-3-phosphate)	−0.417	0.054	0.128
	PEP (Phosphoenol-pyruvate)	−0.113	0.653	0.814
	Pyruvic acid	−0.095	0.473	0.712
	Lactic acid	1.683	<0.0001	<0.0001
TCA cycle	Pyruvic acid	−0.095	0.473	0.712
	Lactic acid	1.683	<0.0001	<0.0001
	Acetyl-CoA	−0.055	0.804	0.866
	Oxaloacetic acid	−0.412	0.241	0.049
	Citric acid	0.070	0.330	0.805
	cis-Aconitic acid	0.884	<0.0001	<0.0001
	Isocitric acid	0.377	0.044	0.078
	2-Ketoglutaric acid	−0.810	<0.0001	<0.0001
	Succinic acid	−0.720	<0.0001	<0.0001
	Fumaric acid	0.079	0.982	0.775
	Malic acid	0.045	0.997	0.854
Glutamine pathway	L-Glutamate	1.266	<0.0001	<0.0001
	L-Glutamine	1.719	<0.0001	<0.0001
	GABA	−0.058	0.171	0.854
choline pathway	Choline	0.731	<0.0001	0.00033
	Phosphocholine	2.218	<0.0001	<0.0001
	Betaine	2.313	<0.0001	<0.0001
Urea cycle	L-Asparate	0.658	0.0005	0.00065
	Fumaric acid	0.079	0.982	0.775
	L-Arginine	1.467	<0.0001	<0.0001
	L-Ornithine	−0.058	0.859	0.854
	L-Citrulline	0.945	<0.0001	<0.0001
	Uric Acid	−0.146	0.131	0.775
Tryptophan cycle	L-Tryptophan	−0.946	<0.0001	<0.0001
	L-Kynurenine	−0.134	0.324	0.643
	Anthranilic acid	−0.287	0.442	0.567
Glutathione cycle	GSH	0.645	0.126	0.078
	GSSG	−0.078	0.677	0.854
	gamma-L-Glutamylcysteine	0.132	0.759	0.742
	Glycine	2.290	<0.0001	<0.0001
	L-Cysteine	0.228	0.213	0.364
purine pathway	Guanine	0.204	0.131	0.406
	Guanosine	1.444	<0.0001	<0.0001
	Hypoxanthine	1.336	<0.0001	<0.0001
	Xanthine	0.097	0.495	0.742
	Adenine	−0.120	0.738	0.805
	Adenosine	0.555	0.012	0.032
Pyrimidine pathway	Cytidine	0.016	0.965	0.941
	Cytosine + Histamine	−0.273	0.239	0.275
	beta-Alanine	1.668	<0.0001	<0.0001
	Thymine	0.034	0.970	0.935
	Uridine	0.981	<0.0001	<0.0001
Amino acid metabolism	Glycine	2.290	<0.0001	<0.0001
	L-Arginine	1.467	<0.0001	<0.0001
	L-Asparate	0.658	0.0005	0.00065
	L-Asparagine	0.472	0.005	0.032
	L-Cysteine	0.228	0.213	0.364
	L-Cystine	−0.298	0.274	0.495
	L-Lysine	1.270	<0.0001	<0.0001
	L-Glutamine	1.719	<0.0001	<0.0001
	L-Glutamate	1.266	<0.0001	<0.0001
	L-Histidine	0.763	<0.0001	<0.0001
	L-Isoleucine	1.575	<0.0001	<0.0001
	L-Leucine	1.366	<0.0001	<0.0001
	L-Methionine	1.064	<0.0001	<0.0001
	L-Phenylalanine	1.640	<0.0001	<0.0001
	L-Proline	1.663	<0.0001	<0.0001
	L-Serine	1.506	<0.0001	<0.0001
	L-Threonine	1.313	<0.0001	<0.0001
	L-Tryptophan	−0.946	<0.0001	<0.0001
	L-Tyrosine	1.270	<0.0001	<0.0001
	L-Valine	1.245	<0.0001	<0.0001

The levels of metabolites in the breast cancer tissue samples and the corresponding normal breast tissue samples were analyzed using LC/MS, and then evaluations based on the glycolytic pathway, TCA cycle, glutamine pathway, choline pathway, urea cycle, tryptophan cycle, glutathione cycle, purine pathway, pyrimidine pathway, and amino acid metabolism were evaluated. The results for each metabolite are shown as A: Log2-fold values for the breast cancer tissue samples vs. the corresponding normal breast tissue samples; B: Log2-fold values for the TN tumors vs. the EP + H- tumors; C: Log2-fold values for the TN tumors vs. the corresponding normal breast tissue samples; and D: Log2-fold values for the EP + H- tumors vs. the corresponding normal breast tissue samples. The Wilcoxon signed-rank test was used for comparisons of metabolite levels between the pairs of breast cancer tissue samples and normal breast tissue samples, and the Mann-Whitney U-test was used for comparison involving the metabolites that were not detected in the paired samples. The false discovery rate (FDR)-adjusted *p* values were also calculated

**Table 3:** Summary of the findings regarding the PC, LPC, PE, and LPE detected in this study

	The number of molecules (Tumor/Normal)			The number of molecules (EP + H−/Normal)			The number of molecules (TN/Normal)			The number of molecules (TN/EP + H-)		
	All	Significant higher	Significant lower	All	Significant higher	Significant lower	All	Significant higher	Significant lower	All	Significant higher	Significant lower
PC	84 (4)	78	4	84 (1)	72	1	84 (1)	74	0	84 (2)	34	0
LPC	43 (36)	31	2	43 (33)	26	4	43 (15)	24	0	43 (19)	1	0
PE	73 (17)	68	2	73 (13)	69	0	73 (11)	62	0	73 (3)	18	1
LPE	22 (13)	19	0	22 (7)	13	0	22 (11)	12	0	22 (15)	0	1

Table 3 shows comparisons of the levels of the PC, LPC, PE, and LPE detected using LC/MS between the breast cancer tissue samples and the corresponding normal breast tissue samples, between the EP + H- tumors and the corresponding normal breast tissue samples, between the TN tumors and the corresponding normal breast tissue samples, and between the TN tumors and the EP + H- tumors. The numbers in parentheses indicate the numbers of PC, LPC, PE, and LPE that were not detected in the paired samples. Significant higher are indicated as follows: tumor > normal, EP + H- > normal, TN > normal, TN > EP + H-, and significant lower are shown as follows: tumor < normal, EP + H- < normal, TN < normal, TN < EP + H- (*p* < 0.05 in both cases). The Wilcoxon signed-rank test was used for comparisons of PC, LPC, PE, and LPE levels between the pairs of breast cancer tissue samples and normal breast tissue samples, and the Mann-Whitney U-test was used for comparisons involving the metabolites that were not detected in the paired samples

### Comparison between the TN and EP + H- tumors

We classified breast cancer tissue samples into TN and EP + H-. The number of TN was 11 and EP + H- was 49. We evaluated the differences in the metabolite profiles of EP + H- and TN, as shown in Table 2B. Most of the metabolites derived from the glycolytic pathway exhibited higher levels in the TN tumors than in the EP + H- tumors. On the other hand, there were no characteristic differences in the levels of metabolites related to the TCA cycle or glutamine cycle between the TN and EP + H- tumors. Regarding the metabolites related to the choline pathway, the levels of choline and phosphocholine in the TN tumors were higher than in the EP + H- tumors, although phosphocholine was only slightly higher. During the immunostaining of choline kinase, we did not observe any prominent differences between the TN and EP + H- tumors (Fig. 2). As for saturated fatty acids, the levels of C16:0 to C28:0 were higher in the TN tumors than in the EP + H- tumors. Regarding unsaturated fatty acids, the levels of the ω3 family C18:3, C18:4 and C20:5 were lower in the TN tumors. The levels of the ω6 family C18:2, C20:3 and C20:4 were lower in TN compared with EP + H- tumors, but the level of C22:4 did not differ. The levels of the ω7 family C16:1 and C18:1 were lower in the TN tumors. The levels of the ω9 family C18:1 and C20:1 were lower in the TN tumors, but the levels of C22:1 and C24:1 were higher (Fig. 3b).

### Comparison between the TN and corresponding normal breast tissue samples (table 2C)

In the levels of glycolytic pathway-related metabolites, the levels of lactic acid and phosphoenol-pyruvate (PEP) were significantly increased between TN and the corresponding normal breast tissue samples, although PEP showed no significant changes in FDR-adjusted *p*-value. Regarding other metabolites, there were no significant differences, and we did not detect any characteristic findings concerning TCA cycle-related metabolites. As for glutamine pathway-related metabolites, the ratio of glutamate to glutamine was higher in the TN tumor samples than in the normal breast tissue samples. Concerning choline pathway-related metabolites, the level of phosphocholine was higher than choline. The levels of arginine and citrulline were significantly higher in the TN tumors, as were glycine, guanosine and hypoxanthine. Most amino acid levels in the TN tumors were also significantly higher. Regarding saturated fatty acids, the level of C16:0 was lower, but the concentrations of C18:0 to C28:0 were higher in the TN tumors than in the normal breast tissue samples. As for unsaturated fatty acids, the levels of ω3 family C18:3 and C18:4 were lower and the level of C20:5 were higher in the normal breast tissue samples. The levels of C18:2 and C18:3 were lower in the TN tumors, but the levels of C20:3, C20:4, and C22:4 were higher. The levels of ω7 family C16:1 and C18:1 were lower in the TN tumors, while that of the ω9 family C18:1 was lower. The levels of C20:1, C22:1 and C24:1 were higher (Fig. 3c).

### Comparison between the EP + H- and corresponding normal breast tissue samples (table 2D)

In the levels of glycolytic pathway-related metabolites, the level of lactic acid was significantly increased between the EP + H- tumor and the corresponding normal breast tissue samples, but no significant differences in other metabolites were detected. No significant differences in the levels of metabolites related to the glycolytic pathway, TCA cycle, other cycles, or amino acid metabolism were detected between the EP + H- tumors and the normal breast tissue samples. However, the analysis of glutamine pathway-related metabolites showed that the levels of both glutamate and glutamine were upregulated in the EP + H- tumors, with the alteration in the level of glutamine being particularly prominent. Regarding saturated fatty acids, the level of C16:0 was lower and those of C18:0 to C24:0 higher in the EP + H- tumors. The levels of C26:0 and C28:0 were lower. As for unsaturated fatty acids, the levels of ω3 family C18:3 and C18:4 were lower and that of C20:5 was higher in normal breast tissue samples. The level of ω6 family C18:2 was lower in the EP + H- tumors, but C20:3, C20:4, and C22:4 were higher. The levels of the ω7 family C16:1 and C18:1 were significantly lower. The level of the ω9 family C18:1 was lower, but the levels of C20:1, C22:1 and C24:1 were significantly higher (Fig. 3d).

### Elongation of saturated and unsaturated fatty acids in the EP + H- and TN tumors

**Fig. 4:**
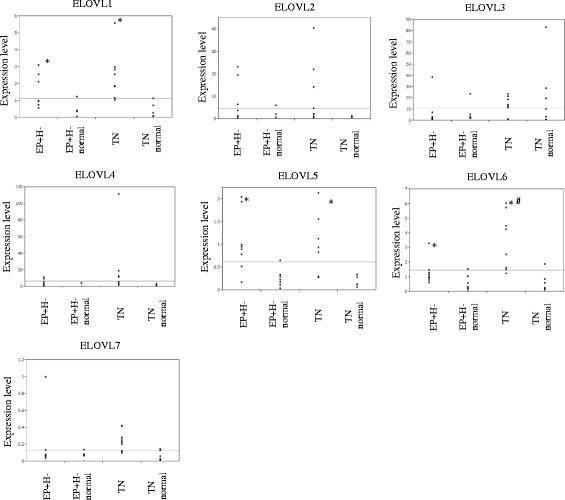
mRNA expressions of elongases in breast cancer and corresponding normal breast tissue. The mRNA expression levels of ELOVL1, 2, 3, 4, 5, 6, and 7 in the TN tumors, EP + H- tumors, and the corresponding normal breast tissue samples were evaluated using real-time RT-PCR. Asterisks indicate significant differences (*p* < 0.05) among the TN tumors, EP + H- tumors, and the corresponding normal breast tissue samples. The sharp sign indicates a significant difference in ELOVL6 expression (*p* < 0.05) between the corresponding TN and EP + H- samples. The Wilcoxon signed-rank test was used for comparisons of the mRNA expression levels of ELOVLs between pairs of breast cancer tissue samples and normal breast tissue samples, and the Mann-Whitney U-test was used for comparisons of the mRNA expression levels of ELOVLs between the TN and EP + H- tumors

**Fig. 5:**
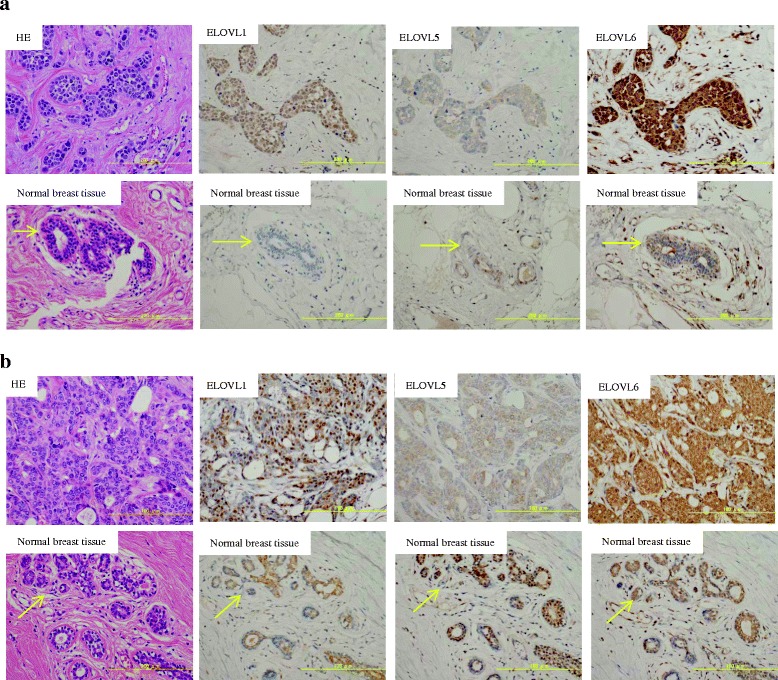
Immunostaining of elongases and HE staining in breast cancer and corresponding normal breast tissues. TN tumors, EP + H- tumors, and the corresponding normal breast tissue samples were subjected to immunostaining of ELOVL1, 5, and 6; nuclear counterstaining with Mayer’s hematoxylin, and HE staining. **a** The upper and lower panels show the results of immunostaining for ELOVL1, 5, and 6 in the TN tumors and the corresponding normal breast tissue samples, respectively. **b** The upper and lower panels show the results of immunostaining for ELOVL1, 5, and 6 in the EP + H- tumors and the corresponding normal breast tissue samples, respectively. The arrows indicate the normal breast tissue samples

As described above, differences in the metabolic pathways associated with saturated and unsaturated fatty acids were observed between each breast cancer subtype (Fig. 3b-d). It is known that the elongases involved in the initial condensation reaction are the rate-limiting enzymes of fatty acid elongation, and 7 types of elongase (ELOVL 1, 2, 3, 4, 5, 6, and 7) are considered to be involved in the ELOVL [29]. To identify the differences in the expression of these ELOVL between the TN tumors and the corresponding normal breast tissue samples, and between the EP + H- tumors and the corresponding normal breast tissue samples, we performed quantitative real-time RT-PCR analyses of ELOVL1–7. As a result, we detected significant changes in the mRNA expression levels of ELOVL1, 5, and 6. The mRNA expression levels of ELOVL1, 5 and 6 were significantly higher by 8.5-, 4.6- and 7.0-fold, respectively, in the TN tumors compared with the corresponding normal breast tissue samples (*p* < 0.05) (Fig. 4). In the EP + H- tumors, the mRNA expression levels of ELOVL1, 5, and 6 were significantly higher by 4.9-, 3.4-, and 2.1-fold, respectively, compared with their levels in the corresponding normal breast tissue samples (*P* < 0.05). In addition, the mRNA expression level of ELOVL6 was a significant 2.6-fold higher in the TN tumors than in the EP + H- tumors (*P* < 0.05). Next, we performed immunostaining studies of ELOVL1, 5, and 6 expression. As a result, ELOVL1 and 6 were more strongly positive in the TN and EP + H- tumors compared with the corresponding normal breast tissue samples (Fig. 5a,b). Furthermore, stronger staining of ELOVL1 and 6 was observed in the TN tumors compared with the EP + H- tumors, although no such phenomenon was seen for ELOVL5.

## Discussion

In this study, the differences in metabolite profiles and pathways between breast cancer tissue samples and the corresponding normal breast tissue samples were evaluated. Then, the breast cancer tissue samples were classified into subtypes, and comparisons between the TN tumors and the corresponding normal breast tissue samples, between the EP + H- tumors and the corresponding normal breast tissue samples, and between the TN and EP + H- tumors were performed. In the analysis of the levels of metabolites related to the glycolytic pathway, the level of lactic acid was significantly increased in breast cancer tissue samples, TN tumors, and H+ tumors, compared with their corresponding normal breast tissue samples. Previous studies reported that glycolysis was upregulated and that the level of lactic acid was significantly increased [9–11, 19, 27, 30]. In our study, there were no significant difference between TN tumors and EP + H- tumors in glycolysis, but the higher level of lactic acid was observed in TN tumors than EP + H- tumors. These results indicate that the glycolytic pathway might be enhanced in TN, as suggested by Pelicano et al. [19]. Inhibition of the glycolytic pathway may, therefore, have an antitumor effect on TN. In the analysis of the levels of metabolites related to the glutamine pathway, significantly higher levels of glutamate and glutamine were observed in the breast cancer tissue samples compared with the normal breast tissue samples, regardless of the cancer subtype. Furthermore, the ratio of glutamate to glutamine was higher in both the EP + H- and TN tumors than in the corresponding normal breast tissue samples. Similar phenomena were reported in a previous study [21], and so significant increases in the levels of glutamate and glutamine, and a high glutamate to glutamine ratio might be useful for distinguishing between breast cancer and normal breast tissue. Furthermore, Budczies et al. reported that the ratio of glutamate to glutamine in ER-negative breast cancer was higher than in ER-positive cancer, but the prognosis was better in patients with a high ratio than those with a low ratio. Therefore, a high glutamate to glutamine ratio may indicate greater susceptibility to chemotherapy [21].

Regarding the levels of metabolites related to the choline pathway, the expression level of choline kinase, which catalyzes the conversion of phosphocholine from choline, is known to be higher in breast cancer tissue than in normal breast tissue [20]. Phosphocholine is used as a base for the production of lipid second messengers and is also the main component of phospholipids and an essential metabolite reservoir for the production of PC [31, 32]. Phosphatidic acid, which is produced from PC, is considered to be the main activator of the mitogen-activated protein kinase (MAPK) and protein kinase B (AKT) signalling pathways [31, 33]. Therefore, choline kinase is required for signaling pathways related to the activation of phosphatidylinositol 3-kinase, AKT, and MAPK [33]. In this study, the level of choline was found to be higher in the breast cancer tissue samples than in the normal breast tissue samples, and moreover, the concentration of phosphocholine was higher than that of choline, suggesting that choline kinase expression is enhanced in breast cancer tissue. It is possible that choline kinase is involved in cancer-related signal transduction [33]. Actually, during immunostaining higher choline kinase expression was detected in the breast cancer tissue samples regardless of the subtype, and these results are supported by the findings of a previous report [33]. The upregulation in the levels of these metabolites are suggested to be dependent on choline kinase, because choline kinase catalyzes the biosynthesis of these metabolites, and in our study, the expression of choline kinase was enhanced in the breast cancer tissues. Other studies also reported that overexpression of choline kinase was found in breast cancer, and this could be the basis for the development of anti-tumor strategies for the breast cancer patients [34–37]. Lipogenesis is considered to be a potential target for treatment of cancer, particularly certain enzymes that are associated with lipogenesis have been reported to be targets for cancer therapy [20]. In our study, higher levels of LPC, LPE, PC, and PE were also detected in the breast cancer tissue samples. In addition to these results, the higher levels of PC and PE in TN tumors were observed in comparison to EP + H- tumors. Hilvo M. et al. reported that levels of phospholipids (eg, phosphatidylinositols, phosphatidylethanolamines, phosphatidylcholines) were increased in breast tumors, and it was significantly higher in ER-negative than ER-positive tumors. Furthermore, their increase levels were positively associated with survival [38].

Differences in fatty acid levels between the cancer subtypes were remarkable. The levels of all saturated fatty acids from C18:0 to C28:0 were higher in the TN tumors than in the corresponding normal breast tissue samples, but the levels of C26:0 and C28:0 were lower in the EP + H- tumors than in the corresponding normal breast tissue samples. The alterations in the levels of these fatty acids are considered to be associated with the elongation of very long chain fatty acids [http://files.webb.uu.se/uploader/271/1819-Singhal-RaviKumar-report.pdf, https://www.diva-portal.org/smash/get/diva2:200324/FULLTEXT01.pdf]. Palmitic acid (C16:0), which is produced by cytoplasmic fatty acid synthesis, and other fatty acids taken from outside cells are extended to produce very long chain fatty acids with long carbon chains. In this reaction, β-ketoacyl synthase is involved in the first step, with β-ketoacyl-CoA synthesized by the condensation of acyl-CoA and malonyl-CoA. This condensation reaction is catalyzed by the elongase enzymes. An elongase, of which 7 types (ELOVL 1, 2, 3, 4, 5, 6 and 7) are described, is involved in the first condensation reaction as a rate-limiting enzyme of fatty acid elongation [29]. Such elongation has been detected in non-alcoholic steatohepatitis (NASH) associated with hepatocellular carcinoma [39]. In squamous cell carcinoma of lung, excessive expression of ELOVL6 was observed, and it was suggested that ELOVL6 inhibition might result in antitumor activity [40]. In addition, the excessive expression of ELOVL6 was found to be related to axillary lymph node metastasis and a short disease-free survival period in breast cancer, and a relationship was suggested to exist between excessive ELOVL6 expression and poor prognosis [41]. Our research detected differences in the synthesis of saturated fatty acids and unsaturated fatty acids between TN and EP + H-. In real-time RT-PCR, higher mRNA expression levels of ELOVL1, 5, and 6 were detected in the TN and EP + H- tumors than in their corresponding normal breast tissue samples, and the mRNA expression level of ELOVL6 in the TN tumors was higher than that seen in the EP + H- tumors. During immunostaining, ELOVL1 and 6 were more strongly stained in the TN and EP + H- tumors than in the corresponding normal breast tissue samples, and furthermore, ELOVL1 and 6 were stained more strongly in the TN tumors than in the EP + H- tumors. Based on these results, it was suggested that fatty acid metabolism pathways involving ELOVL1 and 6 might be targets for therapy against TN.

Currently, TN has no treatment that differs from therapy for EP + H- or HER2 positive breast cancer. However, reports that TN can be classified into multiple subtypes based on its gene expression profile, and it is expected that treatment methods will soon be selected based on the subtype of TN [42–45]. In the future, individualized treatment for TN may be realized through these approaches. From our metabolomics analysis, notable differences in fatty acid metabolism pathways were detected between TN and EP + H-. Therefore, it may be worth exploring inhibition of ELOVL enzymes to treat TN. Furthermore, inhibiting various metabolic pathways, including the glycolytic and glutamine pathways, may also be effective against TN. Limitation of our paper is that the number of samples is small in evaluations for RT-PCR and Immunohistochemistry, because the volume of samples used in this study was limited. In addition, the total number of samples was 74, and the numbers of EP + H- and TN were 49 and 11, respectively. Regarding the remaining 14 samples, the subtype with ER-, PgR+ and HER2- was 3 samples, and the subtype with ER+, PgR- and HER2- was 8 samples, the subtype with and ER+, PgR+ and HER2+ was 3 samples. Therefore, it was difficult to investigate the differences in the metabolite changes between HER+ and HER- and between ER+ and ER-. Second, this study is the pure correlational study without experimental intervention. Therefore, these facts made us conclude that our study has little mechanistic or tumor biological insight, and its reason may be due to the limited number of samples. Taken together, we consider that the further studies using the larger number of samples will be needed in the future, but our findings must be the first and important report about the relationship between breast cancer and ELOVLs.

## Conclusions

We found significant differences between TN and EP + H- tumors in the fatty acid metabolism pathway including greater expression of ELOVL1 and 6. This suggests that the ELOVL1 and 6-related fatty acid metabolism pathway may be a candidate target for therapy of TN.

## Additional files

Additional file 1: Table S1. A list of targeted lipid metabolites in this study. The lipid metabolites targeted in this study were listed in this table. (PDF 14 kb)

Additional file 2: Table S2. A list of targeted cationic metabolites. The cationic metabolites targeted in this study were listed in this table. (PDF 10 kb)

Additional file 3: Table S3. A list of targeted anionic metabolites. The anionic metabolites targeted in this study were listed in this table. (PDF 11 kb)

Additional file 4: Table S4. A list of the cationic and anionic metabolites that were identified by LC/MS analysis. The cationic and anionic metabolites identified by LC/MS analysis of this study were listed in this table. (PDF 16 kb)

Additional file 5: Table S5. A list of the lipid metabolites that were identified by LC/MS analysis. The lipid metabolites identified by LC/MS analysis of this study were listed in this table. (PDF 18 kb)

## Abbreviations

AKT: Protein kinase B
ELOVL: Elongation of very long chain fatty acids
EP + H-: Estrogen receptor-positive, Progesterone receptor-positive, Human epidermal growth factor receptor-negative breast cancers
ER: Estrogen receptor
FISH: fluorescence in situ hybridization
HE: Hematoxylin & eosin
HER2: Human epidermal growth factor receptor-2
LC/MS: Liquid chromatography mass spectrometry
LPC: Lyso-glycerophosphocholines
LPE: Glycerophosphoethanolamines
MAPK: Mitogen-activated protein kinase
NASH: Non-alcoholic steatohepatitis
PC: Glycerophosphocholines
PE: Lyso-glycerophosphoethanolamines
PEP: Phosphoenol-pyruvate
PgR: Progesterone receptor
RT-PCR: Real-time reverse transcription polymerase chain reaction
TCA: Tricarboxylic acid
TN: Triple-negative breast cancer
