# Supplementary material for: Differences in elongation of very long chain fatty acids and fatty acid metabolism between triple-negative and hormone receptor-positive breast cancer
Source: BMC Cancer. 2017 Aug 29;17:589. doi: 10.1186/s12885-017-3554-4 (PMC5576271; doi:10.1186/s12885-017-3554-4)
Supplement: Supplementary file 1 — A list of targeted lipid metabolites in this study. The lipid metabolites targeted in this study were listed in this table. (PDF 14 kb) [file 12885_2017_3554_MOESM1_ESM.pdf]

Supplemental Table 1. A list of targeted lipid metabolites in this study

Compound	Retention time	m/z	Positive/Negative
LPC_14-0 (sn-1)	2.851	468.30>184.10	Positive
LPC_14-0 (sn-2)	3.172	468.30>184.10	Positive
LPC_15-1 (sn-2)	3.25	480.30>184.10	Positive
LPC_16-0p	5.664	480.30>184.10	Positive
LPC_15-0 (sn-1)	3.554	482.30>184.10	Positive
LPC_15-0 (sn-2)	3.915	482.30>184.10	Positive
LPC_16-0e	5.895	482.30>184.10	Positive
LPC_16-1 (sn-1)	3.278	494.30>184.10	Positive
LPC_16-1 (sn-2)	3.605	494.30>184.10	Positive
LPC_16-0 (sn-1)	4.364	496.30>184.10	Positive
LPC_16-0 (sn-2)	4.755	496.30>184.10	Positive
LPC_17-1 (sn-1)	4.003	508.30>184.10	Positive
LPC_17-1 (sn-2)	4.396	508.30>184.10	Positive
LPC_18-0p	7.75	508.30>184.10	Positive
LPC_17-0 (sn-1)	5.385	510.40>184.10	Positive
LPC_17-0 (sn-2)	5.757	510.40>184.10	Positive
LPC_18-3 (sn-1)	3.148	518.30>184.10	Positive
LPC_18-3 (sn-2)	3.413	518.30>184.10	Positive
LPC_18-2 (sn-1)	3.845	520.30>184.10	Positive
LPC_18-2 (sn-2)	4.205	520.30>184.10	Positive
LPC_18-1 (sn-1)	4.838	522.40>184.10	Positive
LPC_18-1 (sn-2)	5.29	522.40>184.10	Positive
LPC_18-0 (sn-1)	6.239	524.40>184.10	Positive
LPC_18-0 (sn-2)	6.742	524.40>184.10	Positive
LPC_19-0 (sn-1)	7.236	538.40>184.10	Positive
LPC_19-0 (sn-2)	7.801	538.40>184.10	Positive
LPC_20-5 (sn-1)	3.095	542.30>184.10	Positive
LPC_20-5 (sn-2)	3.417	542.30>184.10	Positive
LPC_20-4 (sn-1)	3.857	544.30>184.10	Positive
LPC_20-4 (sn-2)	4.214	544.30>184.10	Positive
LPC_20-3 (sn-1)	4.498	546.40>184.10	Positive
LPC_20-3 (sn-2)	4.928	546.40>184.10	Positive
LPC_20-2 (sn-1)	5.481	548.40>184.10	Positive
LPC_20-2 (sn-2)	5.896	548.40>184.10	Positive
LPC_20-1 (sn-1)	6.696	550.40>184.10	Positive
LPC_20-1 (sn-2)	7.17	550.40>184.10	Positive
LPC_20-0 (sn-1)	8.216	552.40>184.10	Positive
LPC_20-0 (sn-2)	8.74	552.40>184.10	Positive
LPC_22-6 (sn-1)	3.844	568.30>184.10	Positive
LPC_22-6 (sn-2)	4.177	568.30>184.10	Positive
LPC_22-4 (sn-1)	5.281	572.40>184.10	Positive
LPC_22-4 (sn-2)	5.66	572.40>184.10	Positive
LPC_22-0 (sn-1)	10.047	580.40>184.10	Positive
LPC_22-0 (sn-2)	10.559	580.40>184.10	Positive
PC_12-0_12-0	9.371	622.40>184.10	Positive
PC_14-0_16-1	13.555	704.50>184.10	Positive
PC_16-0_14-0	13.702	706.50>184.10	Positive
PC_15-0_16-1	13.393	718.50>184.10	Positive
PC_16-0p_16-0	15.315	718.50>184.10	Positive
PC_16-0_15-0	14.026	720.50>184.10	Positive
PC_16-0e_16-0	15.544	720.50>184.10	Positive
PC_14-0_18-2 PC_16-1_16-1	13.167	730.50>184.10	Positive
PC_14-0_18-1 PC_16-0_16-1	13.984	732.50>184.10	Positive
PC_16-0_16-0	14.792	734.60>184.10	Positive
PC_15-0_18-2	13.771	744.60>184.10	Positive
PC_16-0e_18-2	14.961	744.60>184.10	Positive

PC_16-1e_18-1	15.624	744.60>184.10	Positive
PC_15-0_18-1 PC_16-0_17-1	14.488	746.60>184.10	Positive
PC_18-1e_16-0 PC_18-0e_16-1	15.722	746.60>184.10	Positive
PC_17-0_16-0 PC_18-0_15-0	15.76	748.60>184.10	Positive
PC_18-0e_16-0	16.931	748.60>184.10	Positive
PC_14-0_20-5	12.43	752.50>184.10	Positive
PC_16-1_18-3 PC_14-0_20-4	13.11	754.50>184.10	Positive
PC_14-0_20-3	13.762	756.60>184.10	Positive
PC_16-1_18-2 PC_16-0_18-3	13.708	756.60>184.10	Positive
PC_16-0_18-2 PC_16-1_18-1	14.365	758.60>184.10	Positive
PC_16-0_18-1	15.008	760.60>184.10	Positive
PC_16-0_18-0	16.001	762.60>184.10	Positive
PC_15-0_20-5	13.087	766.50>184.10	Positive
PC_16-0e_20-5	14.693	766.50>184.10	Positive
PC_16-0p_20-4	14.654	766.50>184.10	Positive
PC_15-0_20-4	13.732	768.60>184.10	Positive
PC_16-1e_20-3	14.866	768.60>184.10	Positive
PC_17-1_18-2	14.029	770.60>184.10	Positive
PC_18-1e_18-2	15.214	770.60>184.10	Positive
PC_18-2e_18-1	15.988	770.60>184.10	Positive
PC_17-1_18-1 PC_17-0_18-2	14.819	772.60>184.10	Positive
PC_18-0p_18-1 PC_18-1e_18-1	16.225	772.60>184.10	Positive
PC_16-0e_20-2	16.934	772.60>184.10	Positive
PC_17-0_18-1 PC_17-1_18-0 PC_16-0_19-1	15.633	774.60>184.10	Positive
PC_16-1_20-5	12.717	778.50>184.10	Positive
PC_14-0_22-6	13.003	778.50>184.10	Positive
PC_18-2_18-3	13.233	780.60>184.10	Positive
PC_14-0_22-5 PC_16-1_20-4 PC_16-0_20-5	13.635	780.60>184.10	Positive
PC_18-2_18-2 PC_18-1_18-3	13.849	782.60>184.10	Positive
PC_16-0_20-4 PC_16-1_20-3	14.221	782.60>184.10	Positive
PC_18-1_18-2 PC_16-0_20-3 PC_18-0_18-3	14.656	784.60>184.10	Positive
PC_18-1_18-1 PC_18-0_18-2	15.448	786.60>184.10	Positive
PC_18-0_18-1	16.262	788.60>184.10	Positive
PC_16-0p_22-6	14.587	790.60>184.10	Positive
PC_18-0_18-0	18.204	790.60>184.10	Positive
PC_15-0_22-6	13.651	792.60>184.10	Positive
PC_18-1e_20-5	14.53	792.60>184.10	Positive
PC_16-0e_22-6 PC	14.855	792.60>184.10	Positive
PC_17-0_20-5 PC_17-1_20-4	14.022	794.60>184.10	Positive
PC_16-0e_22-5 PC_18-0e_20-5	15.12	794.60>184.10	Positive
PC_18-0p_20-4	15.899	794.60>184.10	Positive
PC_17-0_20-4	14.777	796.60>184.10	Positive
PC_18-1e_20-3	15.64	796.60>184.10	Positive
PC_18-0e_20-4	16.139	796.60>184.10	Positive
PC_17-0_20-3 PC_19-1_18-2	15.201	798.60>184.10	Positive
PC_19-1_18-1 PC_19-0_18-2	16.069	800.60>184.10	Positive
PC_19-0_18-1 PC_18-0_19-1	17.124	802.60>184.10	Positive
PC_18-2_20-5 PC_16-1_22-6	13.207	804.60>184.10	Positive
PC_18-2_20-4 PC_16-0_22-6	14.13	806.60>184.10	Positive
PC_18-1_20-4	14.484	808.60>184.10	Positive
PC_18-0_20-5	14.756	808.60>184.10	Positive
PC_18-1_20-3	14.833	810.60>184.10	Positive
PC_18-0_20-4	15.333	810.60>184.10	Positive
PC_18-1_20-2 PC_18-0_20-3	15.856	812.60>184.10	Positive
PC_16-0_22-2	15.822	814.60>184.10	Positive
PC_18-0_20-2	16.665	814.60>184.10	Positive
PC_20-0_18-1	19.418	816.60>184.10	Positive
PC_17-1_22-6	12.086	818.60>184.10	Positive

PC_18-1e_22-6	14.942	818.60>184.10	Positive
PC_17-0_22-6	14.671	820.60>184.10	Positive
PC_18-1e_22-5	15.642	820.60>184.10	Positive
PC_18-0p_22-5	16.03	820.60>184.10	Positive
PC_19-0_20-3	17.132	826.60>184.10	Positive
PC_20-4_20-4	13.673	830.60>184.10	Positive
PC_20-3_20-4	14.07	832.60>184.10	Positive
PC_18-1_22-6	14.427	832.60>184.10	Positive
PC_20-2_20-4 PC_18-1_22-5	14.864	834.60>184.10	Positive
PC_18-0_22-6	15.254	834.60>184.10	Positive
PC_18-0_22-5	15.642	836.60>184.10	Positive
PC_20-1_20-3 PC_18-0_22-4	16.316	838.60>184.10	Positive
PC_18-1_22-0	15.96	844.70>184.10	Positive
PC_19-0_22-6	15.856	848.60>184.10	Positive
LPE_14-0 (sn-1)	2.929	426.30>285.30	Positive
LPE_14-0 (sn-2)	3.241	426.30>285.30	Positive
LPE_16-0 (sn-1)	4.396	454.30>313.30	Positive
LPE_16-0 (sn-2)	4.848	454.30>313.30	Positive
LPE_17-0 (sn-1)	5.379	468.30>327.30	Positive
LPE_17-0 (sn-2)	5.878	468.30>327.30	Positive
LPE_18-0e	8.029	468.30>327.30	Positive
LPE_18-2 (sn-1)	3.886	478.30>337.30	Positive
LPE_18-2 (sn-2)	4.303	478.30>337.30	Positive
LPE_18-1 (sn-1)	4.946	480.30>339.30	Positive
LPE_18-1 (sn-2)	5.301	480.30>339.30	Positive
LPE_18-0 (sn-1)	6.337	482.30>341.30	Positive
LPE_18-0 (sn-2)	6.752	482.30>341.30	Positive
LPE_20-5 (sn-1)	3.191	500.30>359.30	Positive
LPE_20-5 (sn-2)	3.5	500.30>359.30	Positive
LPE_20-4 (sn-1)	3.935	502.30>361.30	Positive
LPE_20-4 (sn-2)	4.219	502.30>361.30	Positive
LPE_20-3 (sn-1)	4.577	504.30>363.30	Positive
LPE_20-3 (sn-2)	4.948	504.30>363.30	Positive
LPE_20-1 (sn-1)	6.767	508.30>367.30	Positive
LPE_20-1 (sn-2)	7.278	508.30>367.30	Positive
LPE_20-0 (sn-1)	8.373	510.40>369.40	Positive
LPE_20-0 (sn-2)	8.861	510.40>369.40	Positive
LPE_22-6 (sn-1)	3.947	526.30>385.30	Positive
LPE_22-6 (sn-2)	4.266	526.30>385.30	Positive
PE_14-0_18-2	13.171	688.50>547.50	Positive
PE_16-0_16-1 PE_14-0_18-1	13.993	690.50>549.50	Positive
PE_16-0_16-0	14.847	692.50>551.50	Positive
PE_16_1_18_2	14.815	700.50>559.50	Positive
PE_15-0_18-2	13.942	702.50>561.50	Positive
PE_16-0p_18-1	15.908	702.50>561.50	Positive
PE_15-0_18-1	14.675	704.60>563.60	Positive
PE_16-0e_18-1	16.099	704.60>563.60	Positive
PE_16-1_18-2	13.529	714.50>573.50	Positive
PE_16-0_18-3	13.767	714.50>573.50	Positive
PE_16-1_18-1 PE_16-0_18-2	14.329	716.50>575.50	Positive
PE_16-0_18-1	15.053	718.50>577.50	Positive
PE_16-0_18-0	16.419	720.60>579.60	Positive
PE_16-0p_20-5	14.269	722.50>581.50	Positive
PE_16-0p_20-4	14.807	724.50>583.50	Positive
PE_16-0p_20-3 PE_16-0e_20-4	14.899	726.50>585.50	Positive
PE_17-1_18-2	14.143	728.50>587.50	Positive
PE_18-1p_18-1 PE_18-0p_18-2 PE_18-0e_18-3	16.138	728.50>587.50	Positive
PE_17-0_18-2	15.06	730.50>589.50	Positive

PE_18-0e_18-2	16.572	730.50>589.50	Positive
PE_18-0p_18-1	17.537	730.50>589.50	Positive
PE_17-0_18-1	15.925	732.60>591.60	Positive
PE_18-0e_18-1	17.829	732.60>591.60	Positive
PE_16-1_20-5	12.827	736.50>595.50	Positive
PE_18-2_18-3	13.512	738.50>597.50	Positive
PE_16-0_20-5	13.805	738.50>597.50	Positive
PE_18-2_18-2	13.843	740.50>599.50	Positive
PE_16-0_20-4	14.235	740.50>599.50	Positive
PE_18-1_18-2	14.484	742.50>601.50	Positive
PE_18-1_18-1 PE_18-0_18-2	15.696	744.60>603.60	Positive
PE_16-0_20-1 PE_18-0_18-1	16.688	746.60>605.60	Positive
PE_16-0p_22-6	14.792	748.60>607.60	Positive
PE_18-0p_20-5 PE_18-1p_20-4 PE_16-0e_22-6	15.059	750.50>609.50	Positive
PE_17-1_20-4	14.121	752.50>611.50	Positive
PE_18-1p_20-3 PE_16-0p_22-4	15.37	752.50>611.50	Positive
PE_18-0p_20-4	16.206	752.50>611.50	Positive
PE_17-0_20-4	14.946	754.50>613.50	Positive
PE_18-1e_20-3	16.157	754.50>613.50	Positive
PE_18-0e_20-4 PE_20-0e_18-4 PE_20-1e_18-3	16.527	754.50>613.50	Positive
PE_17-0_20-3	15.451	756.60>615.60	Positive
PE_19-0_18-2	16.479	758.60>617.60	Positive
PE_20-0e_18-1	18.919	758.60>617.60	Positive
PE_16-1_22-6 PE_18-2_20-5	13.433	762.50>621.50	Positive
PE_18-2_20-4 PE_18-1_20-5	14.143	764.50>623.50	Positive
PE_16-0_22-6 PE_16-1_22-5 PE_20-2_18-4	14.144	764.50>623.50	Positive
PE_18-1_20-4	14.612	766.50>625.50	Positive
PE_18-0_20-5	14.913	766.50>625.50	Positive
PE_16-0_22-4	14.625	768.60>627.60	Positive
PE_18-1_20-3 PE_18-2_20-2	15.065	768.60>627.60	Positive
PE_18-0_20-4	15.305	768.60>627.60	Positive
PE_20-1_18-2	15.427	770.60>629.60	Positive
PE_18-0_20-3	15.9	770.60>629.60	Positive
PE_18-1_20-1	16.151	772.60>631.60	Positive
PE_20-0_18-2	17.178	772.60>631.60	Positive
PE_18-0_20-1	18.534	774.60>633.60	Positive
PE_17-1_22-6	14.001	776.50>635.50	Positive
PE_18-0p_22-6 PE_18-1p_22-5	15.806	776.50>635.50	Positive
PE_17-0_22-6	14.843	778.50>637.50	Positive
PE_18-0p_22-5 PE_18-1p_22-4	16.362	778.50>637.50	Positive
PE_17-0_22-5	15.234	780.60>639.60	Positive
PE_19-0_20-4	16.267	782.60>641.60	Positive
PE_20-0e_20-4 PE_18-0e_22-4	17.875	782.60>641.60	Positive
PE_18-1_22-6	14.5	790.50>649.50	Positive
PE_18-1_22-5	14.835	792.60>651.60	Positive
PE_18-0_22-6	15.224	792.60>651.60	Positive
PE_18-0_22-5	15.865	794.60>653.60	Positive
PE_18-0_22-4 PE_20-0_20-4	16.692	796.60>655.60	Positive
PE_22-2_18-1 PE_22-1_18-2	17.49	798.60>657.60	Positive
PE_18-1_22-1	18.877	800.60>659.60	Positive
PE_22-0_18-2	19.299	800.60>659.60	Positive
PE_22-0_18-1	21.149	802.60>661.60	Positive
PE_20-2_22-6	14.825	816.60>675.60	Positive
PE_20-1_22-6	15.612	818.60>677.60	Positive
PE_22-1_20-4	17.247	822.60>681.60	Positive
C12:0	2.144	199.00>199.00	Negative
C13:0	2.683	213.20>213.20	Negative
C14:1 (n-5)	2.549	225.20>225.20	Negative

C14:0	3.326	227.10>227.10	Negative
C15:0	4.179	240.80>240.80	Negative
C16:1 (n-7)	3.815	253.10>253.10	Negative
C16:0	5.056	255.05>255.05	Negative
C17:1 (n-7)	4.658	267.20>267.20	Negative
C17:0	6.135	268.90>268.90	Negative
C18:4 (n-3)	2.959	275.20>275.20	Negative
C18:3 (n-3)	3.675	276.90>276.90	Negative
C18:2 (n-6)	4.48	278.95>278.95	Negative
C18:1 (n-9)	5.616	280.90>280.90	Negative
C18:1(n-7)	5.852	280.90>280.90	Negative
C18:0	7.19	283.05>283.05	Negative
C19:0	8.247	296.90>296.90	Negative
C20:5 (n-3)	3.623	300.90>300.90	Negative
C20:4 (n-6)	4.46	303.10>303.10	Negative
C20:3 (n-6)	5.229	305.05>305.05	Negative
C20:2 (n-6)	6.333	307.30>307.30	Negative
C20:1 (n-9)	7.644	309.30>309.30	Negative
C20:0	9.267	311.00>311.00	Negative
C21:0	10.259	325.30>325.30	Negative
C22:6 (n-3)	4.361	326.95>326.95	Negative
C22:5 (n-6)	5.053	329.20>329.20	Negative
C22:4 (n-6)	6.044	331.30>331.30	Negative
C22:1 (n-9)	9.592	337.00>337.00	Negative
C22:0	11.186	339.30>339.30	Negative
C23:0	12.08	353.30>353.30	Negative
C24:1 (n-9)	11.415	365.30>365.30	Negative
C24:0	12.944	367.40>367.40	Negative
C25:0	13.761	381.40>381.40	Negative
C26:0	14.522	395.40>395.40	Negative
C27:0	15.37	409.40>409.40	Negative
C28:0	16.323	423.30>423.30	Negative
4-Cholesten-3-one	13.41	385.30>385.30	Positive
5a-Cholestan-3-one	14.82	387.20>387.20	Positive
Cholic acid	1.31	407.10>407.10	Negative
Glycocholic acid	1.11	464.50>464.50	Negative
Glycodeoxycholic acid	1.44	448.10>448.10	Negative
Taurocholic acid	1.08	514.30>514.30	Negative
Taurochenodeoxycholic acid	1.34	498.50>498.50	Negative
Lithocholic acid	2.54	375.10>375.10	Negative
Chenodeoxycholic acid	1.73	391.30>391.30	Negative
Ursodeoxycholic acid	1.15	391.30>391.30	Negative
Acylcarnitine_C2:0	0.74	204.10>85.05	Positive
Acylcarnitine_C4:0	0.79	233.20>85.05	Positive
Acylcarnitine_C6:0	0.86	261.20>85.05	Positive
Acylcarnitine_C8:0	1.02	289.20>85.05	Positive
Acylcarnitine_C10:0	1	317.30>85.05	Positive
Acylcarnitine_C12:0	1.3	345.30>85.05	Positive
Acylcarnitine_C14:0	2.9	373.30>85.05	Positive
Acylcarnitine_C14:1	2.28	371.30>85.05	Positive
Acylcarnitine_C16:0	4.43	401.40>85.05	Positive
Acylcarnitine_C16:1	3.33	399.30>85.05	Positive
Acylcarnitine_C18:0	6.34	429.40>85.05	Positive
Acylcarnitine_C18:1	4.91	427.40>85.05	Positive
Acylcarnitine_C18:2	3.88	425.40>85.05	Positive
LPE-C16_0p-posi	0.001	438.30>297.30	Positive
LPE-C18_1p-posi	0.001	464.30>323.30	Positive
LPE-C18_0p-posi	0.001	466.30>325.30	Positive

LPE-C22_4-posi	0.001	530.30>389.30	Positive
LPE-C22_1-posi	0.001	536.40>395.40	Positive
LPE-C22_0-posi	0.001	538.40>397.40	Positive
PC-C39_4-posi	0.001	824.60>184.10	Positive
PC-C40_2-posi	0.001	842.70>184.10	Positive
PC-C42_8-posi	0.001	858.60>184.10	Positive
PC-C42_7-posi	0.001	860.60>184.10	Positive
PC-C42_6-posi	0.001	862.60>184.10	Positive
PC-C42_4-posi	0.001	866.70>184.10	Positive
PC-C42_1-posi	0.001	872.70>184.10	Positive
PE-C31_1-posi_17-0_14-1	0.001	676.50>535.50	Positive
PE-C33_0-posi	0.001	706.60>565.50	Positive
PE-C36_0e-posi	0.001	734.60>593.60	Positive
PE-C37_1-posi	0.001	760.60>619.60	Positive
PE-C39_3-posi	0.001	784.60>643.60	Positive
PE-C40_9-posi	0.001	786.50>645.50	Positive
PE-C40_8-posi	0.001	788.50>647.50	Positive
PE-C41_7-posi	0.001	804.60>663.60	Positive
PE-C41_6-posi	0.001	806.60>665.60	Positive
PE-C42_4p-posi	0.001	808.60>667.60	Positive
PE-C42_10-posi	0.001	812.50>671.50	Positive
PE-C42_9-posi	0.001	814.50>673.50	Positive
PE-C42_6-posi	0.001	820.60>679.60	Positive
PE-C44_12-posi	0.001	836.50>695.50	Positive
PE-C44_11-posi	0.001	838.50>697.50	Positive
PE-C44_10-posi	0.001	840.60>699.60	Positive
PE-C46_12-posi	0.001	858.50>717.50	Positive
PE-C46_10-posi	0.001	862.50>721.50	Positive
AC 15_0	0.001	387.30>85.05	Positive
AC 16_2	0.001	397.30>85.05	Positive
AC 17_0	0.001	415.40>85.05	Positive
AC 17_1	0.001	413.40>85.05	Positive
AC 20_0	0.001	457.40>85.05	Positive
AC 20_1	0.001	455.40>85.05	Positive
AC 21_0	0.001	471.40>85.05	Positive
AC 22_0	0.001	485.40>85.05	Positive
AC 23_0	0.001	499.50>85.05	Positive
Glycochenodeoxycholic acid	0.001	448.10>448.10	Negative
