# Supplementary material for: Differences in elongation of very long chain fatty acids and fatty acid metabolism between triple-negative and hormone receptor-positive breast cancer
Source: BMC Cancer. 2017 Aug 29;17:589. doi: 10.1186/s12885-017-3554-4 (PMC5576271; doi:10.1186/s12885-017-3554-4)
Supplement: Supplementary file 2 — A list of targeted cationic metabolites. The cationic metabolites targeted in this study were listed in this table. (PDF 10 kb) [file 12885_2017_3554_MOESM2_ESM.pdf]

Supplemental Table 2. A list of targeted cationic metabolites

Compound	Retention time	m/z	Positive/Negative
Adenine	6.37	135.95>119.00	Positive
Adenosine	12.097	268.00>135.95	Positive
Anthranilic acid	15.54	137.95>64.90	Positive
Betaine	2.046	117.80>58.10	Positive
Betaine aldehyde	2.949	102.00>57.90	Positive
Cadaverine	5.003	103.10>86.05	Positive
DL-3-Aminoisobutyric acid	3.975	103.75>85.90	Positive
Carnitine	4.613	161.80>60.00	Positive
Carnosine	4.024	227.00>109.95	Positive
Choline	3.482	103.90>60.00	Positive
Creatine	3.06	131.80>44.00	Positive
Creatinine	4.216	114.05>43.90	Positive
Cytidine	6.406	244.00>111.95	Positive
Cytosine Histamine	3.242	112.00>95.05	Positive
GABA	3.263	104.00>86.95	Positive
Glycine	1.604	76.00>30.00	Positive
Guanine	5.03	151.95>134.90	Positive
Guanosine	9.046	283.80>152.10	Positive
4-Hydroxy-L-proline	1.747	131.70>85.90	Positive
Hypoxanthine	3.84	136.95>119.05	Positive
L-Alanine Sarcosine	1.96	90.00>43.95	Positive
beta-Alanine_SIM	1.959	90.25>89.95	Positive
L-Arginine	2.461	175.20>70.10	Positive
L-Asparate	1.704	134.00>73.90	Positive
L-Asparagine	1.743	133.00>74.00	Positive
L-Citrulline	1.986	176.00>69.95	Positive
L-Cysteine_MRM	1.899	122.25>58.95	Positive
L-Cysteine_SIM	1.899	122.25>122.05	Positive
L-Lysine	2.597	147.10>84.15	Positive
L-Glutamine	1.852	147.00>83.90	Positive
L-Glutamate	1.96	147.95>83.95	Positive
L-Histidine	2.604	156.00>110.10	Positive
L-Homoserine D-Homoserine	1.903	120.05>73.95	Positive
L-Isoleucine	9.591	132.00>86.00	Positive
L-allo-Isoleucine	9.265	132.00>86.00	Positive
L-Leucine	10.964	132.00>86.00	Positive
L-Norleucine	11.212	132.00>86.00	Positive
L-Methionine	4.559	149.95>55.95	Positive
L-Ornithine	2.381	133.00>70.10	Positive
L-Phenylalanine	13.818	166.00>120.20	Positive
L-Proline	2.317	116.05>70.10	Positive
L-Serine	1.793	106.00>59.90	Positive
L-Threonine	1.905	120.05>56.10	Positive
L-Tryptophan	17.201	204.80>188.15	Positive
L-Tyrosine	10.183	182.00>90.95	Positive
L-Valine	4.081	118.00>72.00	Positive
L-Norvaline	4.373	118.00>72.10	Positive
DMG	1.871	104.05>58.00	Positive
GSSG_MRM	9.786	613.10>355.05	Positive
GSSG_SIM	9.771	613.10>613.10	Positive
Putrescine	4.019	89.10>72.10	Positive
GSH_MRM	4.011	308.00>76.05	Positive

GSH_SIM	4.011	308.00>308.00	Positive
SAH_MRM	12.928	385.05>135.95	Positive
SAH_SIM	12.942	385.05>385.10	Positive
SAM_MRM	5.097	398.85>250.00	Positive
SAM_SIM	5.093	398.85>399.05	Positive
Spermidine	7.975	146.05>72.00	Positive
Spermine	0.001	202.95>112.15	Positive
Thymidine	0.001	265.10>148.90	Positive
Thymine	5.038	126.95>109.90	Positive
Tyramine	14.097	121.00>77.05	Positive
Uracil	0.001	112.30>71.10	Positive
Uridine	4.093	245.00>113.05	Positive
DL-Homocysteine_MRM	2.785	136.00>90.00	Positive
DL-Homocysteine_SIM	2.784	136.00>136.00	Positive
DL-Homocystine_MRM	3.625	268.95>136.15	Positive
DL-Homocystine_SIM	3.629	268.95>268.95	Positive
L-Cystathionine_MRM	1.863	223.00>87.95	Positive
L-Cystathionine_SIM	1.862	223.00>223.00	Positive
L-Cystine_MRM	1.747	240.95>151.85	Positive
L-Cystine_SIM	1.749	240.95>240.85	Positive
gamma-L-Glutamylcysteine_MRM	3.907	250.75>84.05	Positive
gamma-L-Glutamylcysteine_SIM	3.909	250.75>250.95	Positive
L-Cysteinylglycine_MRM	3.501	178.95>75.90	Positive
L-Cysteinylglycine_SIM	3.501	178.95>178.85	Positive
O-Acetyl-L-homo serine	3.583	162.00>102.10	Positive
N-Acetylglycine	2.48	117.95>76.10	Positive
N-Propionylglycine	3.675	131.95>76.15	Positive
N-Isovaleroylglycine	12.601	160.00>76.15	Positive
L-Pyroglutamic acid	3.162	130.25>83.90	Positive
N-tigloylglycine	0.001	100.95>73.10	Positive
N-Acetyl-L-aspartic Acid	2.784	176.00>133.90	Positive
DL-5-Hydroxylysine	2.406	163.00>81.95	Positive
N-Acetyl-L-tyrosine	13.439	224.00>135.95	Positive
Serotonin	16.388	177.00>159.95	Positive
N-acetyl-L-leucine	15.185	174.00>86.00	Positive
Melatonin	18.134	232.75>174.05	Positive
S-Methyl-L-cysteine_MRM	2.395	119.20>73.05	Positive
N,N-Diethylethanolamine	12.576	117.85>43.90	Positive
Xanthine	3.867	152.95>109.90	Positive
Tryptamine	21.887	144.00>116.95	Positive
2'-Deoxyguanosine	11.86	267.80>151.95	Positive
2'-Deoxyadenosine	12.578	252.00>135.95	Positive
5-Methoxytryptamine	20.727	190.80>173.95	Positive
2'-Deoxycytidine	9.111	228.00>112.15	Positive
Allantoin	1.992	158.95>115.40	Positive
Taurine	1.533	126.00>44.10	Positive
N-Acetyl-DL-alanine	3.97	131.95>89.90	Positive
L-Kynurenine	14.048	209.00>191.95	Positive
Trigonelline	2.617	137.65>91.90	Positive
DL-Ethionine	3.583	164.00>56.05	Positive
N-Acetyl-DL-valine	12.711	160.00>72.10	Positive
(-)-Epinephrine	8.31	184.00>166.00	Positive
(+/-)-Norepinephrine	4.292	151.95>106.90	Positive
Allantoic Acid	16.387	176.95>117.00	Positive

O-Acetyl-L-serine	0.001	147.35>89.90	Positive
Phosphocholine	1.638	184.00>86.00	Positive
5-Hydroxy-L-tryptophan	0.001	220.20>204.10	Positive
Acetyl-L-glutamine	2.525	189.00>84.05	Positive
Dopa	6.836	198.00>151.95	Positive
Dopamine	12.178	154.00>137.05	Positive
beta-Glutamic acid	2.701	147.95>87.90	Positive
L-Theanine	3.657	175.00>83.90	Positive
Acetaminophen	12.441	151.95>110.00	Positive
L-2-Aminobutyric acid	1.869	103.80>57.90	Positive
Uric Acid	3.006	168.95>140.90	Positive
(6R,S)-5-Formyl-5,6,7,8-tetrahydrofolic acid	13.497	474.10>327.05	Positive
10-Formylfolic acid	13.711	470.05>294.95	Positive
Folic acid	14.043	442.05>295.15	Positive
N-Acetylneuraminic acid	1.648	310.00>273.95	Positive
L-Methionine sulfone	2.117	182.00>55.95	Positive
2-Bromohypoxanthine	10.405	214.90>134.90	Positive
