# Supplementary material for: Differences in elongation of very long chain fatty acids and fatty acid metabolism between triple-negative and hormone receptor-positive breast cancer
Source: BMC Cancer. 2017 Aug 29;17:589. doi: 10.1186/s12885-017-3554-4 (PMC5576271; doi:10.1186/s12885-017-3554-4)
Supplement: Supplementary file 3 — A list of targeted anionic metabolites. The anionic metabolites targeted in this study were listed in this table. (PDF 11 kb) [file 12885_2017_3554_MOESM3_ESM.pdf]

Supplemental Table 3. A list of targeted anionic metabolites

Compound	Retention time	m/z	Positive/Negative
Glycolic acid	5.902	75.20>47.10	Negative
Lactic acid	7.029	89.20>43.00	Negative
3-Hydroxy-butyric acid	7.304	103.15>59.10	Negative
Maleic acid	11.103	115.20>71.00	Negative
-Hydroxy-2-methyl-butanoic acid (2-Methyl-3-Hydroxybutyric acid)	8.164	117.25>73.05	Negative
-Hydroxy-2-methyl-butanoic acid (2-Methyl-3-Hydroxybutyric acid)	9.585	117.25>73.05	Negative
Benzoic acid	14.135	121.25>77.05	Negative
Mesaconic acid	12.402	129.20>85.10	Negative
Ethyl-malonic acid	11.935	131.15>87.10	Negative
o-Toluic acid	15.249	135.15>91.10	Negative
3-Amino-2,3-dihydrobenzoic acid	1.575	138.25>121.05	Negative
3-Methyl-glutaric acid	12.1	145.15>83.10	Negative
Thiodiglycolic acid	12.745	149.10>105.00	Negative
2-Hydroxy-phenylacetic acid	14.658	151.15>107.05	Negative
3-Hydroxy-3-methyl-glutaric acid	11.612	161.15>99.10	Negative
Uric acid	5.654	167.10>124.00	Negative
DHAP (Dihydroxyacetone phosphate)	8.734	169.10>97.00	Negative
Shikimic acid	5.627	173.15>93.10	Negative
Tricarballylic acid	12.506	175.10>157.05	Negative
1-Heptane-sulfonic acid	18.777	179.15>80.05	Negative
Azelaic acid	15.415	187.20>125.10	Negative
Glucuronic acid	5.158	193.15>113.05	Negative
4-Hydroxy-3-methoxy-mandelic acid	9.938	197.15>137.05	Negative
R5P (Ribose-5-phosphate)	6.834	229.10>97.05	Negative
1-Hydroxy-2-methyl-2-buten-4-yl 4-diphosphate	12.052	261.10>79.10	Negative
N-Acetyl-neuraminic acid	5.837	308.20>87.15	Negative
UMP	9.178	323.15>97.15	Negative
cGMP	10.661	344.15>150.20	Negative
dTDP	13.56	401.20>79.10	Negative
GDP	12.25	442.20>150.10	Negative
10-Formyl-folic acid	12.935	468.30>120.25	Negative
UDP-D-glucose	12.626	565.15>323.20	Negative
NADP	12.894	742.10>620.10	Negative
Pyruvic acid	8.537	87.20>43.05	Negative
Oxalic acid	11.661	89.20>61.05	Negative
2-Hydroxy-isobutyric acid	8.464	103.15>57.10	Negative
Fumaric acid	12.703	115.20>71.05	Negative
3-Hydroxy-3-methyl-butanoic acid (3-Hydroxyisovaleric acid)	10.185	117.25>59.10	Negative
Nicotinic acid	10.502	122.20>78.10	Negative
4-Methyl-2-oxovaleric acid	14.797	129.25>85.10	Negative
2-Hydroxy-isocaproic acid	14.014	131.25>85.10	Negative
p-Hydroxybenzoic acid	10.565	137.10>93.10	Negative
2-Ethylhexanoic acid	20.2	143.20>143.20	Negative
2-Hydroxy-glutaric acid	11.834	147.15>129.05	Negative
4-Hydroxyphenylacetic acid	11.279	151.15>107.05	Negative
Orotic acid	8.228	155.20>111.05	Negative
Phthalic acid (Benzene-1,2-dicarboxylic acid)	14.478	165.10>77.10	Negative
4-Hydroxy-3-methoxy-benzoic acid	11.057	167.15>108.05	Negative
Glycerol-P (1)	7.406	171.10>79.05	Negative
Glycerol-P (2)	8.314	171.10>79.05	Negative
Suberic acid	13.886	173.20>111.10	Negative
2-Isopropyl-malic acid	13.352	175.20>115.10	Negative
4-Hydroxy-phenyl-lactic acid	11.344	181.15>163.10	Negative
Isocitric acid	12.749	191.10>73.05	Negative
3-aminohippuric acid	9.985	193.15>149.15	Negative
Ru5P (L-Ribulose-5-phosphate 4-epimerase)	8.103	229.10>79.10	Negative
G6P (Glucose-6-phosphate)	6.619	259.20>79.10	Negative
S7P (Sedoheptulose 7-phosphate)	7.109	289.15>97.20	Negative
RuBP (Ribulose-1,5-bisphosphate)	12.962	309.10>97.00	Negative
cAMP	11.913	328.15>134.15	Negative
AMP	10.432	346.15>79.20	Negative
UDP	12.979	403.00>158.95	Negative
5,10-Methylene-5,6,7,8-tetrahydro-folic acid	8.745	454.15>281.30	Negative
5-Formyl-5,6,7,8-tetrahydro-folic acid	12.48	472.15>315.25	Negative
ADP-D-glucose	13.171	588.10>346.15	Negative
Oxamic acid	6.281	88.20>44.05	Negative
Phosphoric acid	6.17	97.15>79.00	Negative
Malonic acid	10.84	103.20>59.10	Negative
Levulinic acid	8.667	115.25>71.10	Negative
3-Ethoxypropionic acid	10.844	117.25>45.05	Negative
Citraconic acid	12.179	129.20>85.10	Negative
Oxaloacetic acid	12.912	131.10>87.10	Negative

Malic acid	11.769	133.20>115.05	Negative
3-Hydroxy-benzoic acid	11.801	137.10>93.10	Negative
Octanoic acid (Caprylic acid)	20.732	143.20>143.20	Negative
trans-Cinnamic acid	16.485	147.15>103.10	Negative
3-Hydroxy-phenylacetic acid	12.351	151.15>107.05	Negative
2-Oxoadipic acid	12.474	159.15>59.10	Negative
b-Phenyl-lactic acid	14.7	165.15>147.10	Negative
PEP (Phosphoenol-pyruvate)	13.177	167.15>79.00	Negative
p-Toluenesulfonic acid	14.579	171.10>80.05	Negative
2-Propylglutaric acid	14.879	173.20>129.10	Negative
Hippuric acid	13.705	178.15>77.10	Negative
3,4-Dihydroxy-mandelic acid (1)	8.657	183.15>121.10	Negative
3,4-Dihydroxy-mandelic acid (2)	11.927	183.15>121.10	Negative
Citric acid	12.354	191.10>111.10	Negative
2-Hydroxy-hippuric acid	14.766	194.15>93.10	Negative
Sebacic acid	16.979	201.20>183.15	Negative
Xu5P (D-Xylulose 5-phosphate)	7.947	229.20>79.10	Negative
F6P (Fructose-6-phosphate)	7.159	259.20>97.10	Negative
Retinoic acid	22.935	299.30>255.25	Negative
dTMP	10.254	321.15>195.10	Negative
FBP (Fructose-1,6-bisphosphate)	12.76	339.10>97.00	Negative
IMP	9.456	347.15>79.20	Negative
ADP	12.274	426.20>79.10	Negative
FMN	14.091	455.10>97.10	Negative
cADP-ribose	6.537	540.05>79.10	Negative
GDP-D-glucose	12.648	604.25>362.15	Negative
FAD	15.397	784.15>437.40	Negative
3-Hydroxy-propionic acid	5.709	89.20>59.15	Negative
3-Hydroxy-isobutyric acid	7.224	103.15>73.10	Negative
Glyceric acid	5.876	105.20>75.05	Negative
Succinic acid	11.104	117.20>73.05	Negative
2-Hydroxy-3-methyl-butyric acid (2-Hydroxyisovaleric acid)	11.719	117.25>71.10	Negative
Glutaconic acid (1)	11.858	129.20>85.05	Negative
Glutaconic acid (2)	13.261	129.20>85.05	Negative
Glutaric acid	11.546	131.15>87.15	Negative
Threonic acid	5.76	135.15>75.05	Negative
Acetyl-salicylic acid (1)	13.773	137.10>93.10	Negative
Acetyl-salicylic acid (2)	15.025	137.10>93.10	Negative
2-Ketoglutaric acid	12.256	145.10>101.00	Negative
Tartaric acid, L-(+)- (RG grade)	12.527	149.10>87.10	Negative
Mandelic acid	12.971	151.15>107.05	Negative
Pimelic acid	12.575	159.15>97.10	Negative
2,3-Pyridine-dicarboxylic acid (Quinolinic acid)	12.726	166.10>78.10	Negative
GAP (Glyceraldehyde-3-phosphate)	7.648	169.10>97.00	Negative
cis-Aconitic acid	13.259	173.10>85.10	Negative
Indol-3-acetic acid (1)	13.257	174.15>130.10	Negative
Indol-3-acetic acid (2)	14.716	174.15>130.10	Negative
Caffeic acid	11.469	179.15>135.10	Negative
2-Phospho-gleceric acid	12.796	185.10>78.95	Negative
Quinic acid	5.877	191.15>85.15	Negative
Gluconic acid	5.538	195.15>75.10	Negative
2-C-methyl D-erythritol 4-phosphate	8.319	215.15>79.10	Negative
Dodecanedioic acid	19.653	229.25>211.20	Negative
G1P (Glucose-1-phosphate)	7.219	259.20>241.10	Negative
CMP	8.387	322.15>79.20	Negative
10-Formyl-pteroic acid	10.967	339.20>164.05	Negative
GMP	9.439	362.15>79.20	Negative
Folic acid	13.27	440.15>311.10	Negative
5-Methyl-tetrahydro-folic acid	13.656	456.35>277.25	Negative
ADP-D-ribose	13.189	558.10>346.15	Negative
NAD	8.484	662.20>540.10	Negative
5-Hydroxy-anthranilic acid	8.022	152.15>108.05	Negative
Gallic acid	8.199	169.10>125.00	Negative
Saccharic acid	12.409	209.15>85.25	Negative
Sinapic acid	12.968	223.15>149.15	Negative
c,c-Dimethylallyl pyrophosphate	15.954	245.10>79.10	Negative
F1P (Fructose-1-phosphate)	8.414	259.20>97.10	Negative
6-Phospho-gluconic acid	12.568	275.20>79.10	Negative
Geranyl pyrophosphate	20.001	313.15>79.20	Negative
PRPP (Phosphoribosyl diphosphate)	15.193	389.10>177.20	Negative
CDP	12.616	402.10>79.10	Negative
T6P (Trehalose-6- phosphate)	6.768	421.15>79.20	Negative
ThPP	7.896	423.05>302.15	Negative
dCTP	15.02	466.00>159.00	Negative

dTTP	14.98	481.00>159.00	Negative
CTP	14.946	482.15>159.00	Negative
UTP	15.263	483.05>158.80	Negative
dATP	15.621	490.10>158.80	Negative
ATP	15.454	506.00>158.90	Negative
GTP	16.118	522.00>79.00	Negative
Glyoxylic acid	6.088	73.20>45.10	Negative
E4P (Erythrose 4-phosphate)	7.874	199.10>97.00	Negative
NADH	13.634	664.20>158.90	Negative
NADPH	15.385	744.10>159.00	Negative
CoA	16.331	766.10>766.10	Negative
Acetyl-CoA	16.647	808.10>408.00	Negative
AcAc-CoA	16.43	850.25>766.00	Negative
Succinyl-CoA	16.584	866.10>408.10	Negative
10-Camphorsulfonic acid	15.532	231.15>80.10	Negative
PIPES	6.552	301.15>193.10	Negative
