# Supplementary material for: Differences in elongation of very long chain fatty acids and fatty acid metabolism between triple-negative and hormone receptor-positive breast cancer
Source: BMC Cancer. 2017 Aug 29;17:589. doi: 10.1186/s12885-017-3554-4 (PMC5576271; doi:10.1186/s12885-017-3554-4)
Supplement: Supplementary file 4 — A list of the cationic and anionic metabolites that were identified by LC/MS analysis. The cationic and anionic metabolites identified by LC/MS analysis of this study were listed in this table. (PDF 16 kb) [file 12885_2017_3554_MOESM4_ESM.pdf]

Supplemental Table 4. A list of the cationic and anionic metabolites that were identified by LC/MS analysis

Metabolite	Log2-fold (Breast cancer/Normal breast)	p-value	FDR-adjusted p-value	Detected number in breast cancer tissue samples	Detected number in the corresponding normal breast tissue samples
Lactic acid	1.828	<0.0001	<0.0001	74	74
3-Hydroxy-2-methyl-butanoic acid (2-Methyl-3-Hydroxybutyri	0.391	0.213	0.0093	74	74
Benzoic acid	0.166	0.526	0.327	74	74
Mesaconic acid	1.230	<0.0001	<0.0001	74	74
Ethyl-malonic acid	0.306	0.002	0.156	74	74
3-Methyl-glutaric acid	0.257	0.186	0.154	74	74
2-Hydroxy-phenylacetic acid	0.209	0.019	0.357	74	74
3-Hydroxy-3-methyl-glutaric acid	0.105	0.419	0.660	73	72
Uric acid	-0.115	0.843	0.667	74	74
Azelaic acid	-0.349	<0.0001	0.014	74	74
Glucuronic acid	-0.396	0.021	0.018	74	73
R5P (Ribose-5-phosphate)	-0.110	0.669	0.634	74	74
N-Acetyl-neuraminic acid	0.024	0.755	0.904	74	73
UDP-D-glucose	0.008	0.767	0.962	66	68
Pyruvic acid	-0.058	0.487	0.788	74	73
Oxalic acid	-0.433	0.00020	0.0014	74	74
3-Hydroxy-3-methyl-butanoic acid (3-Hydroxyisovaleric acid	-0.110	0.265	0.621	74	74
4-Methyl-2-oxovaleric acid	-0.427	<0.0001	0.0011	74	74
2-Hydroxy-isocaproic acid	0.331	0.731	0.040	74	74
p-Hydroxybenzoic acid	0.025	0.200	0.909	74	74
2-Ethylhexanoic acid	0.721	0.012	<0.0001	74	74
2-Hydroxy-glutaric acid	-0.069	0.338	0.015	73	74
Orotic acid	0.139	0.316	0.503	74	74
Phthalic acid (benzene-1,2-dicarboxylic acid	1.134	<0.0001	<0.0001	74	74
4-Hydroxy-3-methoxy-benzoic acid	-0.058	0.837	0.788	74	74
Suberic acid	-0.063	0.287	0.818	74	74
2-Isopropyl-malic acid	0.449	0.174	0.011	74	74
UDP	-0.289	<0.0001	0.043	74	74
ADP-D-glucose	0.081	0.635	0.762	59	57
Oxamic acid	-0.108	0.746	0.660	69	69
Phosphoric acid	-0.297	0.097	0.106	74	74
Citraconic acid	1.146	<0.0001	<0.0001	74	74
Oxaloacetic acid	-0.364	0.134	0.031	74	74
Malic acid	0.103	0.436	0.660	74	67
Octanoic acid (Caprylic acid	0.482	0.601	0.0024	74	74
p-Toluenesulfonic acid	0.048	0.319	0.882	73	74
2-Propylglutaric acid	-0.489	<0.0001	<0.0001	74	74
Citric acid	-0.009	0.307	0.962	74	74
Retinoic acid	0.941	<0.0001	<0.0001	74	74
Glyceric acid	-0.296	0.007	0.082	74	74
Succinic acid	-0.954	<0.0001	<0.0001	74	74
2-Hydroxy-3-methyl-butyric acid (2-Hydroxyisovaleric acid	-0.700	<0.0001	<0.0001	74	74
Glutaconic acid	1.156	<0.0001	<0.0001	74	74
Glutaric acid	0.226	0.0058	0.338	74	74
Threonic acid	-0.511	0.0015	0.0018	74	74
Acetyl-salicylic acid	0.054	0.979	0.799	74	70
2-Ketoglutaric acid	-0.791	<0.0001	<0.0001	74	74
Pimelic acid	-0.575	<0.0001	<0.0001	74	74
2,3-Pyridine-dicarboxylic acid (quinolinic acid	-0.239	0.019	0.103	74	74
cis-Aconitic acid	1.028	<0.0001	<0.0001	74	74
Indol-3-acetic acid	-0.682	<0.0001	<0.0001	74	74
Gluconic acid	0.253	0.016	0.275	73	65
F1P (Fructose-1-phosphate)	0.005	0.654	0.977	69	64
Glyoxylic acid	-0.092	0.761	0.718	74	74
NADH	-0.039	0.920	0.884	68	70
NADPH	-0.076	0.541	0.799	53	51
Acetyl-CoA	0.043	0.921	0.902	38	38
Fumaric acid	0.071	0.617	0.787	68	59
Isocitric acid	0.382	0.016	0.036	73	69
G6P (Glucose-6-phosphate)	-0.370	0.065	0.076	63	51
PEP (Phosphoenol-pyruvate)	0.087	0.925	0.829	32	23
F6P (Fructose-6-phosphate)	-0.071	0.837	0.799	62	39
FBP (fructose-1,6-bisphosphate)	0.190	0.470	0.688	17	27
GAP (Glyceraldehyde-3-phosphate)	-0.261	0.163	0.327	39	42
G1P (glucose-1-phosphate)	-0.280	0.261	0.315	44	37
Adenine	-0.027	0.837	0.948	19	28
Adenosine	0.301	0.035	0.200	74	70
Anthranilic acid	-0.313	0.291	0.372	19	21
Betaine	2.256	<0.0001	<0.0001	74	74
Betaine aldehyde	0.120	0.142	0.846	14	24
DL-3-Aminoisobutyric acid	-0.576	<0.0001	<0.0001	72	67
Carnitine	2.205	<0.0001	<0.0001	74	74
Choline	0.913	<0.0001	<0.0001	74	74
Creatine	3.252	<0.0001	<0.0001	74	68
Creatinine	0.286	<0.0001	0.068	74	74
Cytidine	-0.073	0.660	0.787	74	72
GABA	0.109	0.890	0.696	74	72
Glycine	2.388	<0.0001	<0.0001	74	74
Guanine	0.093	0.355	0.688	74	64
Guanosine	1.449	<0.0001	<0.0001	74	72
4-Hydroxy-L-proline	-0.765	<0.0001	<0.0001	74	74
Hypoxanthine	1.448	<0.0001	<0.0001	74	74
L-Alanine Sarcosine	1.672	<0.0001	<0.0001	74	74
beta-Alanine	1.664	<0.0001	<0.0001	74	74
L-Arginine	1.314	<0.0001	<0.0001	74	74
L-Asparate	0.636	<0.0001	<0.0001	74	74
L-Asparagine	0.524	<0.0001	0.0025	74	74
L-Citrulline	0.925	<0.0001	<0.0001	74	74
L-Cysteine	-0.260	0.217	0.150	73	65
L-Lysine	1.203	<0.0001	<0.0001	74	74
L-Glutamine	1.650	<0.0001	<0.0001	74	74
L-Glutamate	1.404	<0.0001	<0.0001	74	74
L-Histidine	0.719	<0.0001	0.00077	74	73
L-Homoserine D-Homoserine	1.315	<0.0001	<0.0001	74	74
L-Isoleucine	1.597	<0.0001	<0.0001	74	74
L-Leucine	1.386	<0.0001	<0.0001	74	74
L-Methionine	1.016	<0.0001	<0.0001	74	72

L-Ornithine	-0.120	0.766	0.660	74	74
L-Phenylalanine	1.493	<0.0001	<0.0001	74	69
L-Proline	1.714	<0.0001	<0.0001	74	74
L-Serine	1.484	<0.0001	<0.0001	74	74
L-Threonine	1.261	<0.0001	<0.0001	74	74
L-Tryptophan	-0.792	<0.0001	<0.0001	74	74
L-Tyrosine	1.271	<0.0001	<0.0001	74	74
L-Valine	1.267	<0.0001	<0.0001	74	74
L-Norvaline	1.309	<0.0001	<0.0001	74	74
DMG	-0.036	0.044	0.889	74	74
GSSG	-0.647	0.030	0.705	41	16
GSH	3.063	0.042	0.017	62	39
SAH	-0.395	0.0034	0.743	72	47
SAM	0.332	0.822	0.692	60	32
Thymine	0.043	0.908	0.910	28	20
Uridine	0.926	<0.0001	<0.0001	74	74
DL-Homocystine	0.642	0.937	0.431	56	44
L-Cystine	0.882	0.0023	0.338	74	74
L-Cysteine	2.158	<0.0001	0.0024	69	38
gamma-L-Glutamylcysteine	-0.337	0.480	0.799	52	27
N-Acetylglycine	0.150	0.410	0.503	53	52
L-Pyroglutamic acid	0.715	0.00070	0.00077	74	74
N-Acetyl-L-aspartic Acid	-0.085	0.902	0.723	73	64
DL-5-Hydroxylysine	-0.195	0.394	0.608	36	21
N-acetyl-L-leucine	0.089	0.538	0.889	13	13
Melatonin	0.055	0.855	0.882	20	19
S-Methyl-L-cysteine	0.453	0.312	0.503	10	11
Xanthine	0.165	0.315	0.447	73	74
2'-Deoxyguanosine	0.152	0.231	0.503	72	62
Allantoin	0.029	0.821	0.910	32	26
Taurine	0.193	0.178	0.408	61	43
N-Acetyl-DL-alanine	2.042	0.0067	<0.0001	71	73
L-Kynurenine	-0.079	0.404	0.754	72	71
Trigonelline	-0.809	0.003	0.005	14	21
(+/-)-Norepinephrine	2.424	<0.0001	0.315	74	61
O-Acetyl-L-serine	-1.214	0.074	0.185	10	12
Phosphocholine	2.784	<0.0001	<0.0001	74	74
beta-Glutamic acid	0.295	0.183	0.070	72	68
L-Theanine	-0.122	0.746	0.660	74	59
Acetaminophen	0.068	0.215	0.867	35	41
L-2-Aminobutyric acid	0.226	0.0004	0.343	74	74
Uric Acid	-0.073	0.185	0.787	74	74
(6R,S)-5-Formyl-5,6,7,8-tetrahydrofolic acid	-0.056	0.666	0.904	18	16
N-Acetylneuraminic acid	0.024	0.598	0.904	74	74
Folic acid	1.357	0.387	0.754	10	9

A total of 142 cationic and anionic metabolites were identified in the breast cancer tissue samples and the corresponding normal breast tissue samples. The levels of each metabolite in the breast cancer tissue samples are shown as log2-fold values relative to their levels in the corresponding normal breast tissue samples. The Wilcoxon signed-rank test was used for comparisons of metabolite levels between the pairs of breast cancer tissue samples and normal breast tissue samples, and the Mann-Whitney U-test was used for comparisons involving the metabolites that were not detected in the paired samples. The false discovery rate (FDR)-adjusted p values were also calculated. Regarding each metabolite detected in this study, the number of detected samples was shown. FDR, false discovery rate.
