# Supplementary material for: Differences in elongation of very long chain fatty acids and fatty acid metabolism between triple-negative and hormone receptor-positive breast cancer
Source: BMC Cancer. 2017 Aug 29;17:589. doi: 10.1186/s12885-017-3554-4 (PMC5576271; doi:10.1186/s12885-017-3554-4)
Supplement: Supplementary file 5 — A list of the lipid metabolites that were identified by LC/MS analysis. The lipid metabolites identified by LC/MS analysis of this study were listed in this table. (PDF 18 kb) [file 12885_2017_3554_MOESM5_ESM.pdf]

Supplemental Table 5. A list of the lipid metabolites that were identified by LC/MS analysis

Metabolite	Log2-fold (Breast cancer/Normal breast)	p-value	FDR-adjusted p-value	Detected number in breast cancer tissue samples	Detected number in the corresponding normal breast tissue samples
LPC_14-0 (sn-1)	1.164	0.00030	0.0012	73	54
LPC_14-0 (sn-2)	1.805	<0.0001	<0.0001	73	65
LPC_15-1 (sn-2)	1.007	<0.0001	<0.0001	12	12
LPC_16-0p	0.057	0.435	0.7437	69	69
LPC_15-0 (sn-1)	1.531	0.00050	0.0045	61	34
LPC_15-0 (sn-2)	2.021	<0.0001	0.0012	73	53
LPC_16-0e	1.964	<0.0001	0.00060	68	34
LPC_16-1 (sn-1)	2.710	<0.0001	0.00020	74	71
LPC_16-1 (sn-2)	1.399	<0.0001	0.00030	71	73
LPC_16-0 (sn-1)	0.396	0.00030	0.0012	74	74
LPC_16-0 (sn-2)	1.132	<0.0001	<0.0001	74	74
LPC_17-1 (sn-1)	2.578	0.00030	0.0782	56	16
LPC_17-1 (sn-2)	1.433	0.0117	0.0382	61	27
LPC_18-0p	-0.529	0.1643	0.1572	14	11
LPC_17-0 (sn-1)	1.985	<0.0001	<0.0001	73	69
LPC_17-0 (sn-2)	1.982	<0.0001	<0.0001	74	6
LPC_18-3 (sn-1)	0.490	0.9681	0.5458	37	25
LPC_18-3 (sn-2)	1.049	0.764	0.5120	23	15
LPC_18-2 (sn-1)	1.582	<0.0001	<0.0001	74	74
LPC_18-2 (sn-2)	-0.768	0.0045	0.0006	72	73
LPC_18-1 (sn-1)	2.491	<0.0001	<0.0001	74	74
LPC_18-1 (sn-2)	1.143	<0.0001	<0.0001	74	74
LPC_18-0 (sn-1)	0.434	<0.0001	0.00046	74	74
LPC_18-0 (sn-2)	1.629	<0.0001	<0.0001	74	74
LPC_19-0 (sn-1)	2.877	<0.0001	0.0146	51	24
LPC_19-0 (sn-2)	1.753	<0.0001	0.0048	71	43
LPC_20-5 (sn-1)	1.116	0.0013	0.00072	71	60
LPC_20-5 (sn-2)	-0.544	0.0625	0.2086	37	54
LPC_20-4 (sn-1)	2.434	<0.0001	<0.0001	74	3
LPC_20-4 (sn-2)	0.140	0.1119	0.7093	56	73
LPC_20-3 (sn-1)	2.997	<0.0001	<0.0001	74	73
LPC_20-3 (sn-2)	1.023	0.00090	0.0060	70	69
LPC_20-2 (sn-1)	2.655	<0.0001	0.00036	71	49
LPC_20-2 (sn-2)	2.272	<0.0001	<0.0001	74	49
LPC_20-1 (sn-1)	2.068	<0.0001	<0.0001	72	54
LPC_20-1 (sn-2)	2.747	<0.0001	<0.0001	74	71
LPC_20-0 (sn-1)	0.587	0.3858	0.3361	39	17
LPC_20-0 (sn-2)	1.759	<0.0001	<0.0001	73	57
LPC_22-6 (sn-1)	2.094	<0.0001	<0.0001	74	71
LPC_22-6 (sn-2)	-0.521	0.0359	0.0279	70	71
LPC_22-4 (sn-1)	2.943	<0.0001	0.0019	68	23
LPC_22-4 (sn-2)	1.056	0.2847	0.2955	35	11
LPC_22-0 (sn-2)	1.031	0.3123	0.5850	30	8
PC_12-0_12-0	0.004	0.4437	0.9997	74	74
PC_14-0_16-1	1.023	<0.0001	<0.0001	74	74
PC_16-0_14-0	2.335	<0.0001	<0.0001	74	74
PC_15-0_16-1	4.231	<0.0001	<0.0001	74	74
PC_16-0p_16-0	0.073	<0.0001	0.2274	74	74
PC_16-0_15-0	2.693	<0.0001	<0.0001	74	74
PC_16-0e_16-0	1.640	<0.0001	<0.0001	74	74
PC_14-0_18-2 PC_16-1_16-1	3.134	<0.0001	<0.0001	74	74
PC_14_0-18_1 PC_16-0_16-1	2.614	<0.0001	<0.0001	74	74
PC_16-0_16-0	1.028	<0.0001	<0.0001	74	74
PC_15-0_18-2	2.610	<0.0001	<0.0001	74	74
PC_16-0e_18-2	0.153	<0.0001	0.2520	74	74
PC_16-1e_18-1	0.164	<0.0001	0.0548	74	74
PC_15-0_18-1 PC_16-0_17-1	2.953	<0.0001	<0.0001	74	74
PC_18-1e_16-0 PC_18-0e_16-1	1.685	<0.0001	<0.0001	74	74
PC_17-0_16-0 PC_18-0_15-0	1.631	<0.0001	<0.0001	74	74
PC_18-0e_16-0	2.019	<0.0001	<0.0001	74	74
PC_14-0_20-5	3.090	<0.0001	<0.0001	74	72
PC_16-1_18-3 PC_14-0_20-4	2.847	<0.0001	<0.0001	74	74
PC_14-0_20-3	1.565	<0.0001	<0.0001	74	74
PC_16-1_18-2 PC_16-0_18-3	1.549	<0.0001	<0.0001	74	74
PC_16-0_18-2 PC_16-1_18-1	0.991	<0.0001	<0.0001	74	74
PC_16-0_18-1	1.306	<0.0001	<0.0001	74	74
PC_16-0_18-0	1.631	<0.0001	<0.0001	74	74
PC_15-0_20-5	2.563	<0.0001	<0.0001	74	73
PC_16-0e_20-5	-0.008	<0.0001	0.7545	74	74
PC_16-0p_20-4	-0.007	<0.0001	0.7545	74	74
PC_15-0_20-4	2.572	<0.0001	<0.0001	74	74
PC_16-1e_20-3	0.468	<0.0001	0.0002	74	74
PC_17-1_18-2	2.285	<0.0001	<0.0001	74	74
PC_18-1e_18-2	0.195	0.3319	0.1124	74	74
PC_18-2e_18-1	-0.443	0.0107	0.0297	74	74
PC_17-1_18-1 PC_17-0_18-2	1.819	<0.0001	<0.0001	74	74
PC_18-0p_18-1 PC_18-1e_18-1	1.606	<0.0001	<0.0001	74	74
PC_16-0e_20-2	0.304	<0.0001	0.0371	74	74
PC_17-0_18-1 PC_17-1_18-0 PC_16-0_19-1	2.566	<0.0001	<0.0001	74	74
PC_16-1_20-5	1.958	<0.0001	<0.0001	74	74
PC_14-0_22-6	2.597	<0.0001	<0.0001	74	74
PC_18-2_18-3	1.083	<0.0001	<0.0001	74	74
PC_14-0_22-5 PC_16-1_20-4 PC_16-0_20-5	1.064	<0.0001	<0.0001	74	74
PC_18-2_18-2 PC_18-1_18-3	1.338	<0.0001	<0.0001	74	74
PC_16-0_20-4 PC_16-1_20-3	1.062	<0.0001	<0.0001	74	74
PC_18-1_18-2 PC_16-0_20-3 PC_18-0_18-3	1.266	<0.0001	<0.0001	74	74
PC_18-1_18-1 PC_18-0_18-2	1.013	<0.0001	<0.0001	74	74
PC_18-0_18-1	2.196	<0.0001	<0.0001	74	74
PC_16-0p_22-6	0.512	<0.0001	<0.0001	74	74
PC_18-0_18-0	0.991	0.0005	0.0048	74	74
PC_15-0_22-6	2.120	<0.0001	<0.0001	74	74
PC_18-1e_20-5	0.387	<0.0001	0.2031	74	74
PC_16-0e_22-6 PC	0.393	<0.0001	0.0002	74	74
PC_17-0_20-5 PC_17-1_20-4	1.963	<0.0001	<0.0001	74	74
PC_16-0e_22-5 PC_18-0e_20-5	0.726	<0.0001	<0.0001	74	74
PC_18-0p_20-4	0.176	0.0498	0.1607	74	74
PC_17-0_20-4	1.531	<0.0001	<0.0001	74	74
PC_18-1e_20-3	2.159	<0.0001	<0.0001	74	74
PC_18-0e_20-4	1.532	<0.0001	<0.0001	74	74
PC_17-0_20-3 PC_19-1_18-2	1.905	<0.0001	<0.0001	74	74
PC_19-1_18-1 PC_19-0_18-2	2.279	<0.0001	<0.0001	74	74
PC_19-0_18-1 PC_18-0_19-1	2.829	<0.0001	<0.0001	74	74
PC_18-2_20-5 PC_16-1_22-6	1.744	<0.0001	<0.0001	74	74
PC_18-2_20-4 PC_16-0_22-6	1.084	<0.0001	<0.0001	74	74
PC_18-1_20-4	1.181	<0.0001	<0.0001	74	74

PC_18-0_20-5	1.148	<0.0001	<0.0001	74	74
PC_18-1_20-3	1.688	<0.0001	<0.0001	74	74
PC_18-0_20-4	0.980	<0.0001	<0.0001	74	74
PC_18-1_20-2 PC_18-0_20-3	2.414	<0.0001	<0.0001	74	74
PC_16-0_22-2	1.604	<0.0001	<0.0001	74	74
PC_18-0_20-2	2.467	<0.0001	<0.0001	74	74
PC_20-0_18-1	1.472	<0.0001	<0.0001	74	74
PC_17-1_22-6	0.374	0.5816	0.6393	12	9
PC_18-1e_22-6	0.940	<0.0001	<0.0001	74	74
PC_17-0_22-6	1.469	<0.0001	<0.0001	74	74
PC_18-1e_22-5	1.440	<0.0001	<0.0001	74	74
PC_18-0p_22-5	1.542	<0.0001	<0.0001	74	74
PC_19-0_20-3	2.019	<0.0001	<0.0001	74	69
PC_20-4_20-4	1.853	<0.0001	<0.0001	74	74
PC_20-3_20-4	2.062	<0.0001	<0.0001	74	74
PC_18-1_22-6	1.589	<0.0001	<0.0001	74	74
PC_20-2_20-4 PC_18-1_22-5	1.705	<0.0001	<0.0001	74	74
PC_18-0_22-6	0.590	<0.0001	<0.0001	74	74
PC_18-0_22-5	1.496	<0.0001	<0.0001	74	74
PC_20-1_20-3 PC_18-0_22-4	2.349	<0.0001	<0.0001	74	74
PC_18-1_22-0	1.633	<0.0001	0.000090	60	63
PC_19-0_22-6	1.531	<0.0001	<0.0001	74	74
LPE_16-0 (sn-1)	1.231	<0.0001	<0.0001	74	74
LPE_16-0 (sn-2)	2.880	<0.0001	<0.0001	74	74
LPE_17-0 (sn-1)	2.728	0.0265	0.1622	36	10
LPE_17-0 (sn-2)	3.251	<0.0001	0.0063	61	21
LPE_18-0e	-0.127	0.7595	0.9229	22	7
LPE_18-2 (sn-1)	2.044	<0.0001	<0.0001	74	74
LPE_18-2 (sn-2)	0.773	0.0742	0.0156	54	59
LPE_18-1 (sn-1)	2.296	<0.0001	<0.0001	74	74
LPE_18-1 (sn-2)	2.946	<0.0001	<0.0001	74	74
LPE_18-0 (sn-1)	1.643	<0.0001	<0.0001	74	74
LPE_18-0 (sn-2)	3.108	<0.0001	<0.0001	74	74
LPE_20-5 (sn-1)	1.314	0.0184	0.0908	36	18
LPE_20-5 (sn-2)	0.980	0.0051	0.0427	12	13
LPE_20-4 (sn-1)	2.040	<0.0001	<0.0001	74	74
LPE_20-4 (sn-2)	1.995	0.759	0.0033	42	55
LPE_20-3 (sn-1)	2.556	<0.0001	<0.0001	74	74
LPE_20-3 (sn-2)	1.731	0.0054	0.0338	48	21
LPE_20-1 (sn-1)	1.505	0.0093	0.0632	40	17
LPE_20-1 (sn-2)	2.863	<0.0001	<0.0001	65	35
LPE_20-0 (sn-2)	2.087	0.1263	0.4431	39	11
LPE_22-6 (sn-1)	1.780	<0.0001	<0.0001	74	74
LPE_22-6 (sn-2)	1.572	0.0077	0.0040	33	29
PE_14-0_18-2	3.781	<0.0001	<0.0001	74	74
PE_16-0_16-1 PE_14-0_18-1	2.176	<0.0001	<0.0001	74	74
PE_16-0_16-0	0.139	0.3795	0.2853	74	74
PE_16_1_18_2	-0.294	0.0127	0.3319	74	74
PE_15-0_18-2	3.029	<0.0001	<0.0001	71	55
PE_16-0p_18-1	0.930	<0.0001	<0.0001	74	74
PE_15-0_18-1	2.840	<0.0001	<0.0001	74	74
PE_16-0e_18-1	1.562	<0.0001	<0.0001	74	74
PE_16-1_18-2	2.966	<0.0001	<0.0001	74	74
PE_16-0_18-3	2.161	<0.0001	<0.0001	74	74
PE_16-1_18-1 PE_16-0_18-2	1.493	<0.0001	<0.0001	74	74
PE_16-0_18-1	1.200	<0.0001	<0.0001	74	74
PE_16-0_18-0	1.009	<0.0001	<0.0001	74	74
PE_16-0p_20-5	-0.321	<0.0001	0.2082	74	74
PE_16-0p_20-4	0.267	<0.0001	0.00073	74	74
PE_16-0p_20-3 PE_16-0e_20-4	1.052	<0.0001	0.00011	74	74
PE_17-1_18-2	2.525	<0.0001	<0.0001	74	74
PE_18-1p_18-1 PE_18-0p_18-2 PE_18-0e_18-3	0.219	0.0627	0.1841	74	74
PE_17-0_18-2	2.266	<0.0001	<0.0001	74	74
PE_18-0e_18-2	1.516	<0.0001	0.00023	74	74
PE_18-0p_18-1	0.823	<0.0001	<0.0001	74	74
PE_17-0_18-1	2.265	<0.0001	<0.0001	74	74
PE_18-0e_18-1	2.333	<0.0001	<0.0001	74	74
PE_16-1_20-5	2.933	<0.0001	0.0046	74	60
PE_18-2_18-3	2.268	<0.0001	<0.0001	74	74
PE_16-0_20-5	1.491	<0.0001	0.0087	74	74
PE_18-2_18-2	1.711	<0.0001	<0.0001	74	74
PE_16-0_20-4	1.164	<0.0001	<0.0001	74	74
PE_18-1_18-2	1.558	<0.0001	<0.0001	74	74
PE_18-1_18-1 PE_18-0_18-2	1.190	<0.0001	<0.0001	74	74
PE_16-0_20-1 PE_18-0_18-1	1.392	<0.0001	<0.0001	74	74
PE_16-0p_22-6	0.943	<0.0001	<0.0001	74	74
PE_18-0p_20-5 PE_18-1p_20-4 PE_16-0e_22-6	0.370	<0.0001	0.0007	74	74
PE_17-1_20-4	2.171	<0.0001	<0.0001	74	65
PE_18-1p_20-3 PE_16-0p_22-4	0.839	<0.0001	<0.0001	74	74
PE_18-0p_20-4	0.043	<0.0001	0.6393	74	74
PE_17-0_20-4	1.909	<0.0001	<0.0001	74	74
PE_18-1e_20-3	1.485	<0.0001	<0.0001	74	74
PE_18-0e_20-4 PE_20-0e_18-4 PE_20-1e_18-3	1.291	<0.0001	<0.0001	74	74
PE_17-0_20-3	2.741	<0.0001	0.0023	71	45
PE_19-0_18-2	2.783	<0.0001	0.0060	74	73
PE_20-0e_18-1	1.645	<0.0001	<0.0001	74	72
PE_16-1_22-6 PE_18-2_20-5	2.732	<0.0001	<0.0001	74	72
PE_18-2_20-4 PE_18-1_20-5	1.548	<0.0001	<0.0001	74	74
PE_18-1_20-4	1.372	<0.0001	<0.0001	74	74
PE_18-0_20-5	1.291	<0.0001	0.0012	74	74
PE_16-0_22-4	1.500	<0.0001	<0.0001	74	74
PE_18-1_20-3 PE_18-2_20-2	1.667	<0.0001	<0.0001	74	74
PE_18-0_20-4	1.304	<0.0001	<0.0001	74	74
PE_20-1_18-2	1.164	<0.0001	<0.0001	74	74
PE_18-0_20-3	2.023	<0.0001	<0.0001	74	74
PE_18-1_20-1	2.213	<0.0001	<0.0001	74	74
PE_20-0_18-2	1.766	<0.0001	<0.0001	74	74
PE_18-0_20-1	1.452	<0.0001	<0.0001	74	74
PE_17-1_22-6	2.423	0.0010	0.0443	28	15
PE_18-0p_22-6 PE_18-1p_22-5	0.767	<0.0001	<0.0001	74	74
PE_17-0_22-6	2.346	<0.0001	<0.0001	74	74
PE_18-0p_22-5 PE_18-1p_22-4	1.471	<0.0001	<0.0001	74	74
PE_17-0_22-5	2.590	<0.0001	0.0005	59	42
PE_19-0_20-4	2.192	<0.0001	<0.0001	74	60
PE_20-0e_20-4 PE_18-0e_22-4	1.684	<0.0001	0.00025	74	68
PE_18-1_22-6	1.327	<0.0001	<0.0001	74	74
PE_18-1_22-5	1.798	<0.0001	<0.0001	74	74
PE_18-0_22-6	1.504	<0.0001	<0.0001	74	74
PE_18-0_22-5	1.875	<0.0001	<0.0001	74	74

PE_18-0_22-4 PE_20-0_20-4	2.223	<0.0001	<0.0001	74	74
PE_22-2_18-1 PE_22-1_18-2	3.315	<0.0001	<0.0001	74	65
PE_18-1_22-1	2.579	<0.0001	0.00011	74	73
PE_22-0_18-2	1.175	0.055	0.1165	54	43
PE_22-0_18-1	0.812	0.1341	0.1860	56	46
PE_20-2_22-6	2.183	<0.0001	0.0015	57	23
PE_20-1_22-6	1.760	<0.0001	<0.0001	74	65
PE_22-1_20-4	2.271	<0.0001	<0.0001	74	74
C12:0	-0.163	0.0027	0.2641	53	53
C13:0	0.315	0.4387	0.6558	23	22
C14:1 (n-5)	0.548	0.6399	0.2429	74	74
C14:0	-0.320	<0.0001	0.1124	74	74
C15:0	0.269	0.3665	0.1847	44	42
C16:1 (n-7)	-0.362	<0.0001	0.4239	74	73
C16:0	-0.454	<0.0001	0.00042	74	74
C17:1 (n-7)	0.013	0.0773	0.9997	73	73
C17:0	0.230	0.2282	0.2520	68	74
C18:4 (n-3)	-0.598	<0.0001	0.0010	29	46
C18:3 (n-3)	-1.270	<0.0001	<0.0001	73	74
C18:2 (n-6)	-0.914	<0.0001	<0.0001	74	74
C18:1 (n-9)	-0.826	<0.0001	0.00062	74	74
C18:1(n-7)	-0.818	<0.0001	0.00076	74	74
C18:0	0.033	0.5819	0.9484	74	74
C19:0	0.045	0.7498	0.9844	30	28
C20:5 (n-3)	0.740	<0.0001	0.0019	74	73
C20:4 (n-6)	1.869	<0.0001	<0.0001	74	74
C20:3 (n-6)	1.954	<0.0001	<0.0001	74	74
C20:2 (n-6)	1.705	<0.0001	<0.0001	74	74
C20:1 (n-9)	0.516	0.0020	0.0106	74	74
C20:0	0.154	0.4767	0.2429	74	74
C21:0	0.463	0.0262	0.0796	45	43
C22:6 (n-3)	0.624	<0.0001	0.00067	74	74
C22:5 (n-6)	0.967	<0.0001	0.000090	74	74
C22:4 (n-6)	2.317	<0.0001	0.000078	74	74
C22:1 (n-9)	0.777	<0.0001	0.0011	74	72
C22:0	0.581	<0.0001	0.00081	74	74
C23:0	0.741	<0.0001	0.0328	74	74
C24:1 (n-9)	2.099	<0.0001	<0.0001	74	74
C24:0	0.428	<0.0001	0.0094	74	74
C25:0	-0.132	0.0011	0.4338	74	74
C26:0	-0.192	0.0011	0.3196	74	74
C27:0	-0.461	0.0031	0.0013	69	72
C28:0	-0.143	0.0191	0.6393	74	74
4-Cholesten-3-one	-1.711	<0.0001	<0.0001	74	74
5a-Cholestan-3-one	-0.394	0.1011	0.0374	22	22
Cholic acid	-0.953	0,2301	0.0731	16	14
Glycocholic acid	0.538	0,9314	0.6461	12	13
Glycodeoxycholic acid	0.295	0.0494	0.8911	21	25
Taurocholic acid	-0.677	-	-	5	4
Taurochenodeoxycholic acic	-0.494	0.5035	0.0519	17	16
Lithocholic acic	0.072	0.2722	0.9676	28	21
Chenodeoxycholic acid	0.334	0.8815	0.9949	11	9
Ursodeoxycholic acid	0.310	0.9273	0.3773	12	11
Acylcarnitine_C2:0	1.887	<0.0001	<0.0001	74	74
Acylcarnitine_C4:0	3.936	<0.0001	0.000077	74	73
Acylcarnitine_C6:0	2.280	<0.0001	<0.0001	74	60
Acylcarnitine_C8:0	0.720	0.8815	0.0609	13	12
Acylcarnitine_C14:0	3.653	<0.0001	0.000060	74	72
Acylcarnitine_C14:1	2.529	<0.0001	<0.0001	74	72
Acylcarnitine_C16:0	3.854	<0.0001	0.0106	74	74
Acylcarnitine_C16:1	2.433	<0.0001	0.000056	74	73
Acylcarnitine_C18:0	4.476	<0.0001	0.0245	74	74
Acylcarnitine_C18:1	2.835	<0.0001	0.0605	74	74
Acylcarnitine_C18:2	2.536	<0.0001	0.0959	74	73

A total of 278 lipid metabolites were identified in the breast cancer tissue samples and the corresponding normal breast tissue samples. The levels of each metabolite in the breast cancer tissue samples are shown as log2-fold values relative to their levels in the corresponding normal breast tissue samples. The Wilcoxon signed-rank test was used for comparisons of metabolite levels between the pairs of breast cancer tissue samples and the normal breast tissue samples, and the Mann-Whitney U-test was used for comparisons involving the metabolites that were not detected in the paired samples. The false discovery rate (FDR)-adjusted p values were also calculated. .Regarding each metabolite detected in this study, the number of detected samples was shown. The levels of taurocholic acid were measured in 5 tumor samples and 4 normal breast tissue samples so no statistical analysis could be performed. LPC, lyso-glycerophosphocholine; PC, glycerophosphocholine; LPE, lyso-glycerophosphoethanolamine; PE, glycerophosphoethanolamine; FDR, false discovery rate.
